# Minimum sample size for developing a multivariable prediction model using multinomial logistic regression

**DOI:** 10.1177/09622802231151220

**Published:** 2023-01-19

**Authors:** Alexander Pate, Richard D Riley, Gary S Collins, Maarten van Smeden, Ben Van Calster, Joie Ensor, Glen P Martin

**Affiliations:** 1 Division of Informatics, Imaging and Data Science, Faculty of Biology, Medicine and Health, 5292 University of Manchester, Manchester Academic Health Science Centre, Manchester, UK; 2 Centre for Prognosis Research, School of Medicine, 4212 Keele University, Staffordshire, UK; 3 Centre for Statistics in Medicine, Nuffield Department of Orthopaedics, Rheumatology and Musculoskeletal Sciences, University of Oxford, Oxford, UK; 4 552380 NIHR Oxford Biomedical Research Centre, John Radcliffe Hospital, Oxford, UK; 5 Julius Center for Health Sciences, University Medical Center Utrecht, Utrecht University, Utrecht, Netherlands; 6 Department of Clinical Epidemiology, Leiden University Medical Center, Leiden, Netherlands; 7 Department of Development and Regeneration, 26657 KU Leuven, Leuven, Belgium; 8 Department of Biomedical Data Sciences, Leiden University Medical Centre, Leiden, Netherlands; 9 EPI-center, 26657 KU Leuven, Leuven, Belgium

**Keywords:** Clinical prediction models, sample size, multinomial logistic regression, shrinkage

## Abstract

**Aims:**

Multinomial logistic regression models allow one to predict the risk of a categorical outcome with > 2 categories. When developing such a model, researchers should ensure the number of participants ( n ) is appropriate relative to the number of events ( $E_{k}$ ) and the number of predictor parameters ( $p_{k}$ ) for each category *k*. We propose three criteria to determine the minimum *n* required in light of existing criteria developed for binary outcomes.

**Proposed criteria:**

The first criterion aims to minimise the model overfitting. The second aims to minimise the difference between the observed and adjusted $R^{2}$ Nagelkerke. The third criterion aims to ensure the overall risk is estimated precisely. For criterion (i), we show the sample size must be based on the anticipated Cox-snell $R^{2}$ of distinct ‘one-to-one’ logistic regression models corresponding to the sub-models of the multinomial logistic regression, rather than on the overall Cox-snell $R^{2}$ of the multinomial logistic regression.

**Evaluation of criteria:**

We tested the performance of the proposed criteria (i) through a simulation study and found that it resulted in the desired level of overfitting. Criterion (ii) and (iii) were natural extensions from previously proposed criteria for binary outcomes and did not require evaluation through simulation.

**Summary:**

We illustrated how to implement the sample size criteria through a worked example considering the development of a multinomial risk prediction model for tumour type when presented with an ovarian mass. Code is provided for the simulation and worked example. We will embed our proposed criteria within the pmsampsize R library and Stata modules.

## 1 Introduction

Clinical prediction models (CPMs) are developed to predict expected health outcomes, such as an individual's probability that a specific disease or condition is present (diagnostic models) or that a specific event will occur in the future (prognostic models).^1,2^ Logistic regression is typically used for developing CPMs to predict a single binary outcome. Often though, healthcare outcomes have multiple levels (multi-category/ polytomous outcomes), such as cancer grade or Likert scales. Then, the natural extension is to use multinomial logistic regression to develop the CPM. Multinomial models have been used to develop CPMs across a range of clinical settings,^3–8^ and it has been argued they should be used to develop prediction models more often.^9^ It has also been shown that multinomial logistic regression is preferred over multiple binary logistic regression when predicting multiple correlated binary outcomes to estimate their joint probability.^10^

An important design aspect when developing any prediction model is ensuring the sample size of the development dataset is appropriate to minimise overfitting and ensure sufficiently precise predictions. Sample size guidance for developing prediction models with continuous, binary and time-to-event outcomes have recently been developed.^11–15^ However, there is a paucity of guidance for multinomial prediction models. Work by de Jong et al.,^16^ highlighted the importance of considering the number of events per predictor for each outcome category when choosing the sample size, and showed that multinomial logistic regression models were susceptible to overfitting when fit in development data of small-to-medium sample size. However, there is no evidence to support events per predictor rules-of-thumb for calculating the required sample size,^13,17^ and more tailored guidance is required.

Therefore, the aim of this study was to extend the existing sample size criteria by Riley et al.,^11,12^ to cater for multinomial logistic regression prediction models predicting nominal polytomous outcomes. The remainder of this paper is structured as follows: the ‘Existing sample size proposal for developing prediction models using binary logistic regression’ section briefly reviews the minimum sample size criterion outlined by Riley et al.^12^ for binary CPMs, and the ‘Extending the sample size formula to multinomial logistic regression’ section uses these as the foundation for our proposed sample size criteria for developing a multinomial logistic regression model. A detailed description of the simulation used to verify one of the proposed sample size criteria is given in Appendix S1. The ‘Practical recommendations for implementing criteria in practice (estimating $R_{CS\_adj}^{2}$ and dealing with large required sample sizes)’ and ‘A worked example of calculating sample size criteria for a multinomial logistic regression model’ sections illustrate and advise on how to implement the proposed criteria in practice. Finally, in the ‘Discussion’ section we summarise the findings.

## 2 Existing sample size proposal for developing prediction models using binary logistic regression

We use the sample size criteria proposed by Riley et al.^12^ as the basis for our extensions into multinomial logistic regression. In this section, we introduce the notation required for our proposals, but refer readers to previous literature^11,12^ for a full discussion.

Consider a binary outcome, $Y_{i}$ ( i=1,…,N ), which takes the value 1 if observation *i* has the outcome and is 0 otherwise. CPMs for such outcomes aim to estimate the probability of $Y_{i}=1$ conditional on a set of *Q* (candidate) predictor parameters, denoted as $X_{qi}$ for q=1,…,Q, collectively in the vector $X_{i}=(X_{1i},\ldots X_{Qi}{)}^{T}$ . Note that predictor parameters refer to the number of coefficients that must be estimated in the model, rather than the number of covariates included in the model. This can be modelled using logistic regression to estimate $P(Y_{i}=1|X_{i})$ , as

(1) $$\log\;\left(\frac{P(Y_{i}=1|X_{i})}{1-P(Y_{i}=1|X_{i})}\right)=\beta_{0}+\beta_{1}X_{1i}+\ldots +\beta_{Q}X_{Qi}\;,$$

where $\beta_{1},\ldots ,\beta_{Q}$ are a set of predictor coefficients (conditional log odds ratios), which are estimated through maximum likelihood estimation to give estimates ${\hat{\beta }}_{1},\ldots ,{\hat{\beta }}_{Q}$ .

The Riley et al. sample size criteria for developing a binary CPM based on equation (1) have three components detailed in Table 1:

**Table 1.** Three component for deriving a minimum sample size for a binary logistic regression model.

**Criterion (i):** targeting the global shrinkage factor to be above a pre-defined threshold
**Criterion (ii)** targeting a small absolute difference in the apparent and adjusted Nagelkerke's $R^{2}$ ( $R_{\mathrm{Nagelkerke}}^{2}$ )^18^
**Criterion (iii)** targeting a precise estimate of overall risk (model intercepts).

We explain these in more detail and introduce the necessary notation for the rest of the manuscript in this section, and extend each criterion to multinomial logistic regression in the “Extending the sample size formula to multinomial logistic regression” section.

### 2.1 Overview of criterion (i): Sample size to target the global shrinkage factor to be above a pre-defined threshold

The first sample size criterion of Riley et al.^12^ assesses overfitting on the multiplicative scale by considering shrinkage of predictor effects. This is when regression coefficients are shrunk towards zero to help mitigate against risk of overfitting. Criterion (i) is based on a global shrinkage factor (S) that is applied to all predictor effects. Specifically, one multiplies ${\hat{\beta }}_{1},\ldots ,{\hat{\beta }}_{Q}$ of equation (1) by *S*, giving,

(2) $$\log\;\left(\frac{P(Y_{i}=1|X_{i})}{1-P(Y_{i}=1|X_{i})}\right)=\alpha^{*}+S({\hat{\beta }}_{1}X_{1i}+\ldots +\beta_{Q}X_{Qi})\;,$$

where $\alpha^{*}$ is the revised intercept to ensure the mean predicted risk matches the mean observed risk.^19^ For the sample size criteria,^12^ the van Houwelingen and Le Cessie's heuristic shrinkage factor ( $S_{VH}$ )^20^ is used to estimate S :

(3) $$S_{VH}=1-\frac{Q}{LR}\;,$$

where *Q* is the number of candidate predictors parameters considered for inclusion prior to any variable selection, and $LR=-2(lnL_{null}-\ln\;L_{model})$ is the likelihood ratio statistic.

Criterion (i) of Riley et al.,^12^ calculates a sample size *n* to target the shrinkage ( $S_{VH}$ ) to be above a pre-specified threshold (commonly taken as 0.9 or above, to target an overfitting of 10% or less, which leads to greater model stability^21,22^). For binary logistic regression, the required sample size to target a shrinkage factor $S_{VH}$ , is calculated as:

(4) $$n=\frac{Q}{(S_{VH}-1)\log\;\left(1-\frac{R_{CS\_adj}^{2}}{S_{VH}}\right)}\;,$$

where $R_{CS\_adj}^{2}$ , is an optimism-adjusted estimate of the Cox-Snell^23^ $R_{CS}^{2}$ .

### 2.2 Overview of criterion (ii): Ensuring small absolute difference in the apparent and adjusted $R_{\mathrm{Nagelkerke}}^{2}$

The second sample size criterion of Riley et al.^12^ is defined to ensure a small difference ( δ ) between the apparent and adjusted Nagelkerke $R^{2}$ . It requires pre-specifying a value for δ that one would tolerate, with small values preferred to improve model stability.^21,22^ For any generalised linear model, Nagelkerke $R^{2}$ is expressed as

(6) $$R_{\mathrm{Nagelkerke}}^{2}=\frac{R_{CS}^{2}}{\max(R_{CS}^{2})}\;,$$

where $R_{CS}^{2}$ could be the apparent or optimism-adjusted estimate of $R_{CS}^{2}$ . The maximum value of $R_{CS}^{2}$ is calculated as $\max(R_{CS}^{2})=\max(R_{CS\_app}^{2})=\max(R_{CS\_adj}^{2})=1-\exp\;\left(\frac{2\ln\;L_{null}}{n}\right)$ , where $\ln\;L_{null}$ is the log-likehood of the intercept-only model. It then follows that

(6) $$R_{\mathrm{Nagelkerke}\_\mathrm{app}}^{2}-R_{\mathrm{Nagelkerk}e_{\mathrm{adj}}}^{2}=\frac{R_{CS\_app}^{2}}{\max(R_{CS}^{2})}-\frac{R_{CS\_adj}^{2}}{\max(R_{CS}^{2})}\leq \delta$$

holds if the required level of shrinkage ( $S_{VH}$ ) is such that:

(7) $$S_{VH}\geq \frac{R_{CS\_adj}^{2}}{R_{CS\_adj}^{2}+\delta \max(R_{CS}^{2})}\;.$$

For binary logistic regression, the sample size for criterion (ii) is then calculated by substituting the minimum $S_{VH}$ that satisfies equation (7) into equation (4).

### 2.3 Overview of criterion (iii): Ensure precise estimate of overall risk

The third sample size criterion of Riley et al.^12^ is to ensure a precise estimate of overall risk. For binary logistic regression, an approximate 95% confidence interval for the estimate of the overall outcome proportion ( $\hat{\theta }$ ) can be expressed as

(8) $$\hat{\theta }\pm 1.96\sqrt{\frac{\hat{\theta }(1-\hat{\theta })}{n}.}$$

Therefore, to target a pre-specified absolute margin of error of δ , the following sample size is required:

(9) $$n={\left(\frac{1.96}{\delta }\right)}^{2}\hat{\theta }(1-\hat{\theta }).$$

The final (minimum) sample size is then taken to be the maximum sample size across criterion (i), (ii) and (iii).

## 3 Extending the sample size formula to multinomial logistic regression

In this section, we extend each of the criteria from the previous section to the situation where the outcome has multiple categories, and we wish to develop a CPM using multinomial logistic regression. As such, hereto we consider an outcome, $Y_{i}$ ( i=1,…,N ), which has *K* nominal categories, where $Y_{i}=k$ for k∈[1,K] if individual *i* has the k th outcome category. All other notation introduced in the ‘Existing sample size proposal for developing prediction models using binary logistic regression’ section remains the same.

### 3.1 Introducing the multinomial logistic regression model and its calibration framework

A multinomial logistic regression model^24^ predicting outcome, $Y_{i}$ , with *K* nominal categories (taking the first category as the reference, without loss of generality), and *Q* the number of candidate predictor parameters in each sub-model *k*, is expressed by the following set of equations (dropping the subscript *i* for brevity):

(10) $$\begin{array}{c} P(Y=1)=\frac{1}{1+\exp\;\left(\beta_{0,2}+\sum_{q=1}^{Q}\;\beta_{q,2}X_{q}\right)+\ldots +\exp\;\left(\beta_{0,K}+\sum_{q=1}^{Q}\;\beta_{q,K}X_{q}\right)\;}\\ P(Y=2)=\frac{\exp\;\left(\beta_{0,2}+\sum_{q=1}^{Q}\;\beta_{q,2}X_{q}\right)}{1+\exp\;\left(\beta_{0,2}+\sum_{q=1}^{Q}\;\beta_{q,2}X_{q}\right)+\ldots +\exp\;\left(\beta_{0,K}+\sum_{q=1}^{Q}\;\beta_{q,K}X_{q}\right)\;}\\ \vdots \\ \vdots \\ P(Y=K)=\frac{\exp\;\left(\beta_{0,K}+\sum_{q=1}^{Q}\;\beta_{q,K}X_{q}\right)}{1+\exp\;\left(\beta_{0,2}+\sum_{q=1}^{Q}\;\beta_{q,2}X_{q}\right)+\ldots +\exp\;\left(\beta_{0,K}+\sum_{q=1}^{Q}\;\beta_{q,K}X_{q}\right)\;} \end{array}$$

which equates to the following K−1 submodels:

(11) $$\ln\left[\frac{P(Y=k)}{P(Y=1)}\right]=\beta_{0,k}+\overset{Q}{\underset{q=1}{\sum }}\;\beta_{q,k}X_{q}\;$$

for k=2,…,K , alongside the constraint $\overset{K}{\underset{k=1}{\sum }}\;P(Y=k)=1$ .

Sub-model specific shrinkage factors can be defined for a multinomial logistic regression based on the recalibration framework outlined by Van Hoorde et al.^25^ Specifically, after fitting a multinomial logistic regression using maximum likelihood (equation (11)), a separate shrinkage factor $S_{MN,k}$ is applied to all the β 's for each sub-model *k*, and each intercept updated (to ensure calibration-in-the-large), as follows:

(12) $$\ln\left[\frac{P(Y=k)}{P(Y=1)}\right]=\alpha_{0,k}^{*}+S_{MN,k}\left(\overset{Q}{\underset{q=1}{\sum }}\;{\hat{\beta }}_{q,k}X_{q}\right)$$

for k=2,…,K , where ${\hat{\beta }}_{m,k}$ are the maximum likelihood estimates from the multinomial logistic regression (equation (11)), and $\alpha_{0,k}^{*}$ are the re-estimated intercepts.

### 3.2 Extending criterion (i) to multinomial logistic regression

#### Direct application of criterion (i) to multinomial logistic regression models

A natural starting point for criterion (i) from binary logistic regression to multinomial logistic regression, would be to again base the required sample on targeting the single heuristic shrinkage factor of van Houwelingen and Le Cessie^20^ to be at or above the chosen threshold. If we let

(13) $$S_{VH\_MN}=1-\frac{(K-1)*Q}{LR_{MN}}\;,$$

be the heuristic shrinkage factor of the multinomial model, where $LR_{MN}=-2(lnL_{null}-lnL_{model})\;$ is like likelihood ratio test statistic for the multinomial model, (K−1)*Q is the total number of candidate predictor parameters across all sub-models, and $R_{CS\_adj}^{2}=1-\exp\left(\frac{-LR_{MN}}{n}\right)$ is the apparent estimate of the Cox-Snell generalised definition of $R^{2}$ for the multinomial model, then equation (4) could again be used to define a minimum required sample size to target $S_{VH\_MN}$ to be at a pre-specified threshold:

(14) $$n=\frac{(K-1)*Q}{(S_{VH\_MN}-1)\ln\left(1-\frac{R_{CS\_adj}^{2}}{S_{VH\_MN}}\right)}\;.$$

However, this approach has issues. While the van Houwelingen and Le Cessie^20^ heuristic shrinkage factor is an estimator of the (true) *S* for binary logistic regression, there is no clear relationship between $S_{VH\_MN}$ and the K−1 multinomial sub-model specific shrinkage factors $\left(S_{MN,k}:\;k=2,\ldots ,K\right)$ in equation (12). Therefore, simply ensuring that $S_{VH\_MN}$ surpasses a pre-specified threshold (using equation (14)) would not necessarily result in the required level of shrinkage in each sub-model (i.e. $S_{MN,k}:\;k=2,\ldots ,K$ in equation (12)). This is undesirable because it would mean that some sub-models of the multinomial model could be overfit. Therefore, we propose another approach to extend criterion (i), which targets all sub-model shrinkage factors $\left(S_{MN,k}:k=2,\ldots ,K\right)$ to be at or above the desired threshold.

#### Alternative suggestion for criterion (i), utilising distinct logistic regression models

Equation (11) can be expressed as a set of K−1 distinct logistic regression models fitted separately, in the subset of the cohort which has either outcome *k* or outcome 1 (the reference). That is, the following binary logistic regression model can be fitted,

(15) $$\ln\left[\frac{P(Y=k)}{P(Y=1)}\right]=\gamma_{0,k}+\overset{Q}{\underset{q=1}{\sum }}\;\gamma_{q,k}X_{q},$$

on the subset of individuals where Y∈{1,k} , separately for k=2,3,…,K . These are also referred to as ‘one vs one’ models.^6^

Crucially, a separate shrinkage factor for each distinct logistic regression model can then be calculated,^26–28^ such that

(16) $$\ln\left[\frac{P(Y=k)}{P(Y=1)}\right]=\gamma_{0,k}^{*}+S_{DL,k}\left(\overset{Q}{\underset{q=1}{\sum }}\;{\hat{\gamma }}_{q,k}X_{q}\right),$$

where ${\hat{\gamma }}_{m,k}$ are the coefficients estimated from equation (15), $\gamma_{0,k}^{*}$ are the re-estimated intercepts, and $S_{DL,k}$ is the shrinkage factor for distinct logistic regression model *k*, defined in the same way as *S* from the ‘Overview of criterion (i): Sample size to target the global shrinkage factor to be above a pre-defined threshold’ section (now referred to as ‘distinct logistic shrinkage factors’). This means that if an estimate of $R_{CS\_adj}^{2}$ is available for each distinct logistic regression model *k*, then using the process summarised in the ‘Overview of criterion (i): Sample size to target the global shrinkage factor to be above a pre-defined threshold’ section, equation (4) can be used to derive a sample size to target a particular distinct logistic shrinkage factor for each model. Importantly, it has been shown that the sub-models of the multinomial logistic regression, and the distinct logistic regression models, are parametrically equivalent ( $\gamma_{m,k}=\beta_{m,k}$ ).^29^ Given the asymptotically unbiased property of the maximum likelihood estimators, it follows that ${\hat{\gamma }}_{m,k}={\hat{\beta }}_{m,k}$ as N→∞ , and hence $S_{DL,k}\to S_{MN,k}$ as N→∞ . Therefore, deriving a sample size to target the shrinkage factor of each distinct logistic regression ( $S_{DL,k}:\;k=2,\ldots ,K$ ) is above the desired value using equation (4),^12^ will also target the multinomial sub-model specific shrinkage factors ( $S_{MN,k}:\;k=2,\ldots ,K$ ) to be above the desired threshold. A separate sample size calculation must therefore be done for each pair of outcomes, taking the maximum to ensure criterion (i) is satisfied for each sub-model. One further point, this strategy relies on the fact that $S_{DL,k}\to S_{MN,k}$ as N→∞ . For the sample size criteria to work, we need close agreement between $S_{DL,k}$ and $S_{MN,k}$ at the value of *N* that satisfies the sample size criteria. We therefore report the agreement between $S_{DL,k}$ and $S_{MN,k}$ in the simulation carried out in the supplementary material, at a sample size *N* that meets said criteria.

#### Consideration of the choice of reference category

So far, the first outcome category has been taken as the reference. During model development, changing the reference category will not have an impact on the risk scores generated from the model. However, upon validating a multinomial CPM, the choice of reference category will change which of the multinomial sub-model specific shrinkage factors are calculated (i.e. what is estimated from equation (12)). Therefore, for the purposes of criterion (i), one must ensure that the shrinkage of every possible sub-model across all reference categories at model validation is above a certain level. Not doing so (i.e. only focusing on one outcome category), would potentially create over-confidence in the model's ability to distinguish between some of the outcome categories. In other words, one must aim to minimise optimism in all pairwise performance metrics. While calculating criterion (i) with taking each outcome category as a reference in turn may lead to high required sample sizes (e.g. see the ‘A worked example of calculating sample size criteria for a multinomial logistic regression model’ section), this would be reflective of one trying to develop a CPM to predict multinomial outcomes that require a lot of statistical power; this should be viewed as valuable information rather than a hindrance (as with any sample size calculation). We, therefore, outline our final approach in the following section, to ensure overfitting is minimised across all pairs of outcomes.

#### Final proposal for criterion (i)

Let $S_{MN,k,r}$ be the sub-model specific shrinkage factor from the van Hoorde et al.^25^ framework, for sub-model *k* with the reference category *r*. The following approach will target every $S_{MN,k,r}$ to be above the pre-specified threshold. The proposal is to follow the approach outlined in the ‘Alternative suggestion for criterion (i), utilising distinct logistic regression models’ section for every possible reference category, and take the maximum sample size across all reference categories. That is for each distinct logistic regression model {k,r} where k≠r ,

(17) $$\ln\left[\frac{P(Y=k)}{P(Y=r)}\right]=\gamma_{0,k,r}+\overset{Q}{\underset{q=1}{\sum }}\;\gamma_{q,k,r}X_{q},$$

we can obtain a corresponding shrinkage factor, $S_{DL,k,r}$ , each defined in the same way as $S_{DL,k}$ from equation (16); that is,

(18) $$\ln\left[\frac{P(Y=k)}{P(Y=r)}\right]=\gamma_{0,j,r}^{*}+S_{DL,k,r}\left(\overset{Q}{\underset{q=1}{\sum }}\;{\hat{\gamma }}_{q,k,r}X_{q}\right).$$

Define $m_{k,r}$ as the number of individuals with outcome category *k* or *r* that is required to target the shrinkage factor $S_{DL,k,r}$ to be above some pre-defined threshold (e.g. 0.9). Here, $m_{k,r}$ can be calculated using the existing formula for binary logistic regression^12^; specifically, using equation (4). To do so, appropriate estimates of $R_{CS,k,r}^{2}$ (Cox-Snell $R^{2}$ for distinct logistic regression model {k,r} ), *Q* (the number of candidate predictor parameters considered for inclusion in each sub-model), and $p_{k}$ (the proportion of individuals from the cohort expected to have outcome k ) must each be pre-specified. Suggestions of how to pre-specify $R_{CS,k,r}^{2}$ are given in the ‘Practical recommendations for implementing criteria in practice (estimating $R_{CS\_adj}^{2}$ and dealing with large required sample sizes)’ section. Note that because the logistic regression models for $\ln\left[\frac{P(Y=k)}{P(Y=r)}\right]$ and $\ln\left[\frac{P(Y=r)}{P(Y=k)}\right]$ are equivalent, we can reduce this to only consider the combinations where k>r . 

The total number of individuals required in the whole cohort ( $n_{k,r}$ ) to ensure there are $m_{k,r}$ individuals with outcome categories {k,r} , can then be calculated as $n_{k,r}=m_{k,r}/p_{k,r}\;$ , where $p_{k,r}$ is the proportion of individuals from the whole cohort expected to have outcome categories {k,r} . Finally, the required sample size *n* to satisfy our criterion (i) is taken to be $n=\max(n_{k,r}:\;k>r)$ .

The proposed approach to implementing criterion (i) are evaluated in a simulation study with full details provided in Appendix S1.

### 3.3 Extending criterion (ii) to multinomial logistic regression

As noted in the ‘Overview of criterion (ii): Ensuring small absolute difference in the apparent and adjusted $R_{\mathrm{Nagelkerke}}^{2}$ ’ section, the second criterion outlined by Riley et al.,^12^ is defined to ensure a small difference (δ) between the observed and expected proportion of variance explained ( $R_{\mathrm{Nagelkerke}}^{2}$ ) for the overall model.

As outlined in de Jong et al.,^16^ the apparent $R_{\mathrm{Nagelkerke}}^{2}$ for a multinomial logistic regression model is defined in the same as for a binary logistic model:

(19) $$R_{\mathrm{Nagelkerke}}^{2}=\frac{1-\exp\left(\frac{-LR_{MN}}{n}\right)}{1-\exp\left(\frac{2lnL_{null}}{n}\right)}=\frac{R_{CS}^{2}}{\max(R_{CS\_app}^{2})}\;\;,$$

where $LR_{MN}$ is defined as previously, and $lnL_{null}$ is the log-likelihood of an intercept only multinomial model. Therefore, given the definition of $S_{VH\_MN}$ in equation (13), to ensure a difference of less than δ between the apparent and adjusted $R_{\mathrm{Nagelkerke}}^{2}$ the following equation must hold:

(20) $$S_{VH\_MN}\geq \frac{R_{CS\_adj}^{2}}{R_{CS\_adj}^{2}+\delta \max(R_{CS\_app}^{2})}\;,$$

Plugging this into equation (14), and noting that *n* is a monotonically increasing function of $S_{VH\_MN}$ , we get the following requirement for criterion (ii) for multinomial logistic regression prediction models:

(21) $$n\geq \frac{(K-1)*Q}{\left(\frac{R_{CS\_adj}^{2}}{R_{CS\_adj}^{2}+\delta \max(R_{CS\_app}^{2})}-1\right)\ln\;(1-R_{CS\_adj}^{2}-\delta \max(R_{CS\_app}^{2}))}\;,$$

Given $R_{\mathrm{Nagelkerke}}^{2}$ is similarly defined for binary logistic and multinomial logistic regression models, this criterion is directly transferable from binary logistic regression to multinomial models. In line with the criterion (the ‘Overview of criterion (ii): Ensuring small absolute difference in the apparent and adjusted $R_{\mathrm{Nagelkerke}}^{2}$ ’ section) for binary logistic regression, we recommend a difference of δ = 0.05.^11,12^

We note that for criterion (ii), we focus on the fit of the overall multinomial logistic regression model, in contrast to criterion (i) where we focused on each sub-model. The reason for this is that $R_{CS}^{2}$ (and hence $R_{\mathrm{Nagelkerke}}^{2}$ ) is not typically expressed for the sub-models of a multinomial logistic regression. While we could ensure that criterion (ii) holds for each distinct logistic regression model, it is not clear what this would achieve with respect to the sub-models of the multinomial logistic regression model.

### 3.4 Extending criterion (iii) to multinomial logistic regression

As outlined in the ‘Overview of criterion (iii): Ensure precise estimate of overall risk’ section, the third criterion of Riley et al.,^12^ is to ensure a precise estimate of overall risk (i.e. model intercept). To mimic the approach for binary logistic regression, for a multinomial model, this can be approximated by calculating the margin of error in the outcome proportion estimates.

Let $p_{k}=E_{k}/n$ be the proportion of individuals from the entire cohort with outcome category {k} , with $E_{k}$ the number of events in outcome category *k*. If $\pi_{k}$ is the underlying multinomial probability of outcome category *k*, then it can be shown through the work of Quesenberry and Hurst,^30^ and Goodman,^31^ that the simultaneous α×100 % confidence interval limits for $\pi_{1},\pi_{2},\ldots \pi_{K}$ :

$$\pi_{k}^{-}\leq \pi_{k}\leq \pi_{k}^{+};k=1,\ldots ,K$$

can be estimated by

(22) $$p_{k}\pm {\left[\chi_{\frac{\alpha }{K},1}^{2}\times \frac{\;p_{k}(1-p_{k})}{n}\right]}^{\frac{1}{2}}$$

where $\chi_{\frac{\alpha }{K},1}^{2}$ denotes the Chi-squared distribution with 1 degree of freedom. Therefore, the sample size to ensure an absolute margin of error δ (say 0.05) at a (1−α)×100 % confidence level is

(23) $$n=\underset{k=1,\ldots ,K}{\max}\;\left(\frac{\chi_{\frac{\alpha }{K},1}^{2}\times p_{k}(1-p_{k})}{\delta^{2}}\right).$$

We choose to target simultaneous confidence intervals^30,31^ rather than pointwise confidence intervals so that every estimate of overall risk will simultaneously be within the pre-defined margin of error. This will require a larger sample size than considering pointwise confidence intervals and is therefore conservative.

It is important to mention that we are primarily interested in a precise estimate of the mean risk of each outcome category across all individuals in the population after adjustment for predictors. However, the mean risk of each outcome category across all individuals will often be similar to the outcome proportions observed from a null model with no predictors (which we are working with above). The variability of these two quantities will therefore also be similar, and we can approximate the variability of the mean risk of each outcome category in the population using the above formula.

A summary of our proposed sample size criteria for a multinomial logistic regression model is given in Table 2.

**Table 2.** Summary of the proposed minimum sample size criteria for multinomial logistic regression CPMs

**Step 1: Choose number of predictor parameters** Q **considered for inclusion in each sub-model at model development**
Recognise that one predictor may require > 1 predictor parameter; for example, categorical predictor with > 2 categories, a continuous predictor with nonlinear terms, and interaction terms.
**Step 2: Choose sensible values for** $p_{k}$ **and** $p_{k,r}$ **, the proportion of individuals in the cohort with outcomes in category** *k* **and {*k, r*}** $max(R_{CS\_app}^{2})$ **and** $R_{CS\_adj}^{2}$ **for the multinomial model, and** $R_{CS\_adj,k,r}^{2}$ **of each distinct logistic regression model**
Ideally, this will be based on previously published models in the same setting with similar outcome definition, a variety of ways to estimate these from various reported statistics are given in the ‘Practical recommendations for implementing criteria in practice (estimating $R_{CS\_adj,k,r}^{2}$ and dealing with large required sample sizes)’ section. If no previous information is available to estimate $R_{CS\_adj,k,r}^{2}$ , use values which correspond to an $R_{\mathrm{Nagelkerke}}^{2}=0.15$ in each sub-model.
**Step 3: Criterion (i)**
1. Calculate the minimum sample size ( $m_{k,r}$ ) for each distinct logistic regression model {k,r} , where k>r , using equation (4) based on a pre-specified level of shrinkage (for example, targeting shrinkage factors of 0.9 ) and an estimate of $R_{CS\_adj,k,r}^{2}$ .
2. Calculate the total number of individuals needed to achieve the required number in each distinct logistic regression model {k,r} , by dividing by $p_{k,r},$ $n_{k,r}=m_{k,r}/p_{k,r}$
3. Take the minimum sample size for criterion (i) to be $n=max(n_{k,r}:\;k>r)$ , which will target all the multinomial sub-model specific shrinkage factors to be greater-than-or-equal to the pre-specified threshold.
**Step 4: Criterion (ii)**
Use equation (21) to calculate a sample size to target the difference between the apparent and optimism adjusted $R_{\mathrm{Nagelkerke}}^{2}$ to be δ , using estimates of $\max(R_{CS\_app}^{2})$ and $R_{CS\_adj}^{2}$ . Previously δ=0.05 has been recommended.^14^
**Step 5: Criterion (iii)**
Use equation (23) to calculate a sample size to target the simultaneous 95% confidence intervals of the estimates of overall risk for each category to be ≤δ , using estimates of $p_{k}$ . We recommend δ=0.05 .
**Step 6: Final sample size**
The required minimum sample size is the maximum value from steps 3 to 5, to ensure criteria (i), (ii) and (iii) are met.

## 4 Practical recommendations for implementing criteria in practice (estimating $R_{CS\_adj}^{2}$ and dealing with large required sample sizes)

To perform our proposed sample size calculations, an estimate of $R_{CS\_adj}^{2}$ needs to be pre-specified. As with earlier work^11,12,14^ we recommend that this is based on similar, previously developed or validated prediction models. When calculating criterion (i), estimates of $R_{CS\_adj,k,r}^{2}$ are required for each distinct logistic regression model {k,r} , corresponding to the sub-models of the multinomial logistic model. When calculating criterion (ii) an estimate of $R_{CS\_adj}^{2}$ is required for the multinomial logistic regression model. We discuss how to estimate these using the published data below. We also urge researchers to report the metrics discussed below when publishing future CPM development papers based on multinomial logistic regressions, to aid the sample size calculations of others. Finally, we make recommendations on what to do if the calculated required sample size is unfeasibly high.

### 4.1 Recommendations for deriving $R_{CS_{adj},k,i}^{2}$ of distinct logistic regression models

To calculate criterion (i) estimates of $R_{CS\_adj,k,r}^{2}$ are required. If the appropriate ‘one-vs-one’^6^ distinct logistic regression models have been fitted in a published study and estimates of $R_{CS\_adj,k,r}^{2}$ have been reported, these can be used directly. If other pseudo- $R^{2}$ statistics have been reported (for each distinct logistic), there are a variety of ways to derive $R_{CS\_adj,k,r}^{2}$ from these; see Riley et al.^12^ Alternatively, if the C-statistics of each distinct logistic regression model are available, then $R_{CS\_adj,k,r}^{2}$ can be estimated using a simulation approach.^15^

However, it is highly likely that each distinct logistic regressions will not have been fitted alongside any previously developed multinomial logistic regression model. In this case, the pairwise C-statistics^32^ (using the conditional risk method) of the multinomial logistic regression might have been reported. Here, since these pairwise C-statistics provide an estimate of the C-statistic for each distinct logistic regression model, they can be used to estimate $R_{CS\_adj,k,r}^{2}$ using the simulation approach of Riley et al.^15^ We illustrate this approach in our worked example in the ‘A worked example of calculating sample size criteria for a multinomial logistic regression model’ section.

If neither pseudo- $R^{2}$ or (pairwise) C-statistics are available a priori, we suggest calculating the minimum sample size following the approach suggested by Riley et al.,^14^ for when information on $R_{CS\_adj}^{2}$ is not available. Specifically, under a conservative assumption of optimism adjusted $R_{\mathrm{Nagelkerke}}^{2}$ of 0.15 (15%), equation (5) can be modified to give $R_{CS\_app,k,r}^{2}=0.15*\max(R_{CS\_app,k,r}^{2})$ for each distinct logistic regression model. Here, $\max(R_{CS\_app,k,r}^{2})$ can be estimated for each model using equation (6):

(24) $$\max(R_{CS\_app,k,r}^{2})=1-\exp\;\left(\frac{2*lnL_{null,k,r}}{n}\right)\;,$$

where $\ln L_{null,k,r}$ can be calculated for each distinct logistic regression model using:

(25) $$lnL_{null,k,r}=E_{k}\ln\left(\frac{E_{k}}{E_{k}+E_{r}}\right)+E_{r}\ln\left(\frac{E_{r}}{E_{k}+E_{r}}\right)\;,$$

where $E_{k}$ and $E_{r}\;$ are the number of outcome events in the category *k* and *r*, respectively. Alternatively (and equally), for each distinct logistic regression model $\max(R_{CS\_app,k,r}^{2})$ can be calculated as:

(26) $$\max(R_{CS\_app,k,r}^{2})=1-(\varphi_{k,r}^{\varphi_{k,r}}{\left(1-\varphi_{k,r}\right)}^{1-\varphi_{k,r}}{)}^{2},$$

where $\varphi_{k,r}=E_{k}/E_{k}+E_{r}$ , is the outcome proportion in the category *k* relative to the reference category *r*. If a multinomial model had been published, then this information would be available for each distinct logistic regression model assuming the number of events in each category had been reported.

### 4.2 Recommendations for deriving $R_{CS\_adj}^{2}$ of multinomial logistic regression models

To calculate criterion (ii) a pre-specified estimate of the overall $R_{CS\_adj}^{2}$ is required. As previous, this would ideally be based on information from a previous multinomial logistic regression model. Similarly to binary logistic regression, if other pseudo- $R^{2}$ statistics have been reported, there are a variety of ways to derive $R_{CS\_adj}^{2}$ from these, as outlined in Riley et al.^12^

Alternatively, one could again take a conservative approach to setting $R_{CS\_adj}^{2}=0.15*\max(R_{CS\_app}^{2})$ (corresponding to an $R_{\mathrm{Nagelkerke}}^{2}$ of 0.15 ). There are two ways to calculate $\max(R_{CS\_app}^{2})$ for multinomial logistic regression. The first is to use equation (6), where $\ln L_{null}$ can be calculated for a multinomial logistic regression as:

(27) $$\ln L_{null}=\overset{K}{\underset{k=1}{\sum }}\;E_{k}\ln\left(\frac{E_{k}}{n}\right),$$

with $E_{k}$ denoting the number of events in outcome category *k*. Alternatively, $\max(R_{CS\_app}^{2})$ can be expressed as:

(28) $$\max(R_{CS\_app}^{2})=1-{\left(\overset{K}{\underset{k=1}{\prod }}\;{\left(\;p_{k}\right)}^{\;p_{k}}\right)}^{2},$$

where $p_{k}=E_{k}/n$ is the observed frequency of category *k*, as defined in the ‘Extending criterion (iii) to multinomial logistic regression’ section. This expression follows naturally from equations (6) and (27), and details of its derivation are given in Appendix S1. Some implications of basing the estimate of $R_{CS\_app}^{2}$ on the assumption $R_{\mathrm{Nagelkerke}}^{2}=0.15$ are also given in Appendix S1.

### 4.3 Recommendations if required sample size is too high

We propose three strategies if the required sample size is completely unfeasible to recruit. It is worth reiterating, that the estimated sample size is required to build the proposed model with the specified levels of overfitting, optimism and precision. In order to reduce the sample size, the model must either be simplified, or you must be willing to accept overfitting, optimism and precision below the desired level.

1. Merge outcome categories. We believe the first consideration could be to merge some of the outcome categories that are driving the high sample size; looking at each pairwise criterion (i) will indicate which categories are driving the sample size. This should only be done if it makes sense from a clinical point of view, and knowing the risks of the merged categories would be of clinical interest.
2. A second suggestion is to reduce the number of candidate predictor parameters considered for inclusion in the model, which is inline with previous suggestions.^11,12,14^ However, we have only looked at scenarios where there are a fixed number of predictor parameters considered for each sub-model. This means when reducing the number of predictor parameters, one would be doing so across all sub-models. An alternative to this is to only reduce the number of predictor parameters considered for inclusion in the sub-model(s) with the highest level of overfitting. This is an enticing approach, as one does not want to reduce the number of predictor parameters in sub-models that are not suffering from overfitting. However, the implications of such an approach are not yet clear and would require further research before this could be recommended.
3. Reduce the acceptable level of overfitting between specific pairs of outcomes. Rather than having the acceptable level of shrinkage at 0.9, it could be reduced (e.g. to 0.8), specifically for the pair of outcomes that are driving the high sample size. This is somewhat undesirable as criterion (i) is in place to minimise overfitting. However, at least the targeted level of overfitting would be explicitly stated and the limitations of the model would therefore be well quantified.

## 5 A worked example of calculating sample size criteria for a multinomial logistic regression model

### 5.1 Hypothetical scenario and information available in literature

In this section, we present a worked example to illustrate how our proposed sample size criteria could be implemented in practice. The code that was used to do this is available on GitHub.^33^ Our example aims to calculate the minimum sample size required to develop a multinomial logistic regression prediction model to predict the tumour type (benign, borderline, stage I invasive, stage II-IV invasive, or metastatic) when presented with an ovarian mass. This is an important preoperative diagnosis, as dependent on the type of tumour, different clinical action may be taken.

Van Calster et al.^8^ considered the development of such a model using the International Ovarian Tumor Analysis Group^34^ dataset. The following information is available from that work. The model was developed on a dataset of 3506 tumours, of which 2557 were benign, 186 were borderline, 176 were stage I invasive, 467 were stage II–IV invasive, and 120 were metastatic. The following pairwise C-statistics^32^ were reported for every combination of outcome comparisons: 0.85 (benign vs borderline), 0.92 (benign vs stage I invasive), 0.99 (benign vs stage II–IV invasive), and 0.95 (benign vs metastatic), 0.75 (borderline vs stage I invasive), 0.95 (borderline vs stage II–IV invasive), 0.87 (borderline vs metastatic), 0.87 (stage I invasive vs stage II–IV invasive), 0.71 (stage I invasive vs metastatic) and 0.82 (stage II–IV invasive vs metastatic). These pairwise C-statistics are reported from a temporal validation and are free from in-sample optimism concerns, therefore we can use these to estimate $R_{CS\_adj,k,i}^{2}$ directly with no adjustment for optimism required. There were 17 candidate predictor parameters considered for inclusion in the model including all the fractional polynomials of continuous variables (each extra fractional polynomial term counts as an additional predictor parameter). We will assume we will consider the same set of variables for inclusion before applying variable selection techniques. We now illustrate the use of the aforementioned information to perform our sample size calculation.

### 5.2 Steps 1 and 2: Identifying values for $p_{k}$ , $p_{k,r}$ , $\max(R_{CS\_app}^{2})$ , $R_{CS\_adj}^{2}$ , and $R_{CS\_adj,k,r}^{2}$

k and *r* take the values 1 (benign), 2 (borderline), 3 (stage I invasive), 4 (stage II–IV invasive), and 5 (metastatic).

#### Calculating Q

Assuming we consider the same set of variables for variable selection that were used in the work by Van Calster et al.,^8^ this would mean Q=17 .

#### Calculating $p_{k}$ and $p_{k,r}$

$p_{k}$ is the proportion of individuals that have outcome category ∈{k} , $p_{k,r}$ is the proportion of individuals that have outcome category ∈{k,r} where k>r . To estimate these values, we use the prevalence of each outcome category as reported in the ‘Hypothetical scenario and information available in literature’ section: $p_{1}=0.729$ , $p_{2}=0.053$ , $p_{3}=0.050$ , $p_{4}=0.133$ , $p_{5}=0.034$ , $p_{2,1}=0.782$ , $p_{3,1}=0.780$ , $p_{4,1}=0.863$ , $p_{5,1}=0.764$ , $p_{3,2}=0.103$ , $p_{4,2}=0.186$ , $p_{5,2}=0.087$ , $p_{4,3}=0.183$ , $p_{5,3}=0.084$ , $p_{5,4}=0.167$ .

#### Calculating $\max(R_{CS\_app}^{2})$

We calculated $\max(R_{CS\_app}^{2})$ using equation (28), and the prevalence of each outcome category $p_{k}$ :

$$\max(R_{CS_{app}}^{2})=1-(0.729^{0.729}*0.053^{0.053}*0.050^{0.050}*0.133^{0.133}*0.034^{0.034}{)}^{2}=0.841.\;$$

#### Calculating $R_{CS\_adj}^{2}$

Given the $R_{CS\_adj}^{2}$ of the overall multinomial model had not been reported, we based our estimate of $R_{CS\_adj}^{2}$ on assuming $R_{\mathrm{Nagelkerke}}^{2}=0.15$ . Using the estimate of $\max(R_{CS\_app}^{2})$ in equation (5) gave an estimate of:

$$R_{CS\_adj}^{2}=0.15*\max(R_{CS_{app}}^{2})=0.15*0.841=0.126.$$

#### Calculating $R_{CS\_adj,k,r}^{2}$

No data was available on the $R_{CS\_adj,k}^{2}$ in the work of Van Calster et al.,^8^ therefore we had to estimate them indirectly using the simulation approach of Riley et al.^15^ This method utilisises the pairwise C-statistics, on which data was available.^8^ Estimates of the pairwise outcome proportions $\varphi_{k,r}$ of category *k* relative to the reference category *r* are also required to implement the simulation approach. These were estimated using the number of tumours in each category: $\varphi_{2,1}=0.068$ , $\varphi_{3,1}=0.064$ , $\varphi_{4,1}=0.154$ , $\varphi_{5,1}=0.045$ , $\varphi_{3,2}=0.486$ , $\varphi_{4,2}=0.715$ , $\varphi_{5,2}=0.392$ , $\varphi_{4,3}=0.726$ , $\varphi_{5,3}=0.405$ , $\varphi_{5,4}=0.204$ . The simulation approach was followed for each sub-model to give estimates of: $R_{CS\_adj,2,1}^{2}=0.116$ , $R_{CS\_adj,3,1}^{2}=0.179$ , $R_{CS\_adj,4,1}^{2}=0.497$ , $R_{CS\_adj,5,1}^{2}=0.170$ , $R_{CS\_adj,3,2}^{2}=0.185$ , $R_{CS\_adj,4,2}^{2}=0.499$ , $R_{CS\_adj,5,2}^{2}=0.374$ , $R_{CS\_adj,4,3}^{2}=0.328$ , $R_{CS\_adj,5,3}^{2}=0.129$ , $R_{CS\_adj,5,4}^{2}=0.210$ . Code for this simulation approach is provided at the GitHub repsitory,^33^ as well as more general code on how to implement this simulation approach in the original work.^15^

### 5.3 Step 3: Criterion (i)

Following the process in the ‘Final proposal for criterion (i)’ section, first, each $m_{k,r}$ was calculated using equation (4) and the estimates of $R_{CS\_adj,k,r}^{2}$ from the ‘Calculating $R_{CS\_adj,k,r}^{2}$ ’ section. Then the total number of individuals required to target a multinomial sub-model specific shrinkage factor of 0.9 for sub-model {k,r} , $n_{k,r}$ , was calculated by dividing $m_{k,r}$ by $p_{k,r}$ :

$$\begin{array}{c} n_{2,1}=m_{2,1}/p_{2,1}=\frac{17}{(0.9-1)\ln\;\left(1-\frac{0.116}{0.9}\right)}*\frac{3506}{2557+186}=1574\\ n_{3,1}=m_{3,1}/p_{3,1}=\frac{17}{(0.9-1)\ln\;\left(1-\frac{0.179}{0.9}\right)}*\frac{3506}{2557+176}=982\\ n_{4,1}=m_{4,1}/p_{4,1}=\frac{17}{(0.9-1)\ln\;\left(1-\frac{0.497}{0.9}\right)}*\frac{3506}{2557+467}=246\\ n_{5,1}=m_{5,1}/p_{5,1}=\frac{17}{(0.9-1)\ln\;\left(1-\frac{0.170}{0.9}\right)}*\frac{3506}{2557+120}=1067\\ n_{3,2}=m_{3,2}/p_{3,2}=\frac{17}{(0.9-1)\ln\;\left(1-\frac{0.185}{0.9}\right)}*\frac{3506}{186+176}=7147\\ n_{4,2}=m_{4,2}/p_{4,2}=\frac{17}{(0.9-1)\ln\;\left(1-\frac{0.499}{0.9}\right)}*\frac{3506}{186+467}=1128\\ n_{5,2}=m_{5,2}/p_{5,2}=\frac{17}{(0.9-1)\ln\;\left(1-\frac{0.374}{0.9}\right)}*\frac{3506}{186+120}=3629\\ n_{4,3}=m_{4,3}/p_{4,3}=\frac{17}{(0.9-1)\ln\;\left(1-\frac{0.328}{0.9}\right)}*\frac{3506}{176+467}=2045\\ n_{5,3}=m_{5,3}/p_{5,3}=\frac{17}{(0.9-1)\ln\;\left(1-\frac{0.129}{0.9}\right)}*\frac{3506}{176+120}=13063\\ n_{5,4}=m_{5,4}/p_{5,4}=\frac{17}{(0.9-1)\ln\;\left(1-\frac{0.210}{0.9}\right)}*\frac{3506}{467+120}=3813 \end{array}$$

The minimum required sample size was taken as the maximum of these, and therefore N=13063 , approximately 9527 benign tumours, 693 borderline, 656 stage I invasive, 1740 stage II–IV invasive and 447 metastatic (assuming same outcome proportions as in Van Calster et al.^8^).

### 5.4 Step 4: Criterion (ii)

Criterion (ii) aims to calculate a sample size required to ensure a difference of 0.05 between the apparent and adjusted $R_{\mathrm{Nagelkerke}}^{2}$ , which holds if equation (21) is satisfied (‘Extending criterion (ii) to multinomial logistic regression’ section). Plugging in the estimates of $\max(R_{CS\_app}^{2})$ and $R_{CS\_adj}^{2}$ into equation (21) gives:

$$n\geq \frac{4*17}{\left(\frac{0.126\;}{0.126\;+0.05*0.841\;}-1\right)\ln\;(1-0.126-0.05*0.841)}$$

n≥1477.

So 1477 individuals are required to meet criterion (ii), approximately 1077 benign tumours, 78 borderline, 74 stage I invasive, 197 stage II–IV invasive, and 51 metastatic.

### 5.5 Step 5: Criterion (iii)

Criterion (iii) is to ensure a precise estimate of risk in the overall population. Following the steps outlined in the ‘Extending criterion (iii) to multinomial logistic regression’ section, for a 95% confidence interval ( α=0.05) , using the estimated values for $p_{k},$ with K=5 and an absolute margin of error of δ=0.05 , then the required sample size for each outcome is (equation (23)):

$$n_{1}=\frac{6.635\times 0.729(1-0.729)}{0.05^{2}}=524$$

$$n_{2}=\frac{6.635\times 0.053(1-0.053)}{0.05^{2}}=134$$

$$n_{3}=\frac{6.635\times 0.50(1-0.150)}{0.05^{2}}=127$$

$$n_{4}=\frac{6.635\times 0.133(1-0.133)}{0.05^{2}}=307$$

$$n_{5}=\frac{6.635\times 0.034(1-0.034)}{0.05^{2}}=88\;$$

Leaving the sample size for criterion (iii) to be $\max(n_{1},\;n_{2},n_{3},n_{4},n_{5})=524,$ approximately 382 benign tumours, 28 borderline, 26 stage I invasive, 70 stage II–IV invasive, and 18 metastatic.

### 5.6 Step 6: Final sample size

The final sample size is taken as the maximum of the sample sizes required to satisfy each criterion (i)–(iii), which was 13,063 (i), 1476 (ii) and 524 (iii) respectively. Hence, the minimum required sample size would be n=13063 , approximately 9527 benign tumours, 693 borderline, 656 stage I invasive, 1740 stage II–IV invasive and 447 metastatic (assuming same outcome proportions as in Van Calster et al.^8^). For contrast, using the definition of events per variable (EPV_m_) from De Jong et al.,^16^ an EPV_m_ of 10 would result in a sample size of 4967 , and an EPV_m_ of 20 would result in a sample size of 9934 .

### 5.7 Suggestions for dealing with high sample size

The required sample size is high and is being driven by outcome categories 3 (stage I invasive) and 5 (metastatic). If the proposed model was developed with a sample size smaller than 13,063, the level of overfitting between these two outcomes would not be targeted at the pre-specified level of 0.9 . Following the suggestions in the ‘Recommendations if required sample size is too high’ section, the first solution would be to merge categories 3 and 4 (stage I invasive with stage II–IV invasive). With such a combination the model would retain clinical interpretation. If it was essential to keep these outcome categories separate, fewer predictor parameters could be considered instead. The value of Q=17 incorporates fractional polynomial terms and interactions which could be removed, or one of the predictors could be removed altogether. A final possible option is to reduce the targeted level of overfitting for pair {3,5} . Plugging a value of 0.8 into the ‘Step 3: Criterion (i)’ section would give $n_{5,3}=5746$ , and the final sample size would be driven by $n_{3,2}=7147$ . While this is slightly undesirable, the targeted level of overfitting for all other outcome pairs would still be at 0.9 , and one could report that overfitting may be more likely for outcome pair {3,5} .

## 6 Discussion

We have presented sample size criteria for the development of prediction models for multiple-category outcomes using multinomial logistic regression. This builds upon recent developments in this space for continuous, binary and time-to-event outcomes.^11,12^ Criterion (ii) and (iii) both had a natural extension into a multinomial framework. Criterion (i) did not and therefore we tested the properties of our proposed approach in a simulation (Appendix S1), finding that the sample size resulted in the desired level of overfitting. Our approach to criterion (i) may lead to high sample sizes if some of the outcome categories are rare, or have a low pairwise $R_{CS}^{2}$ , however this is necessary if you want to ensure overfitting is minimised in prediction between all pairs of outcome categories. If the required sample size cannot be achieved, we have made some recommendations on how the model could be adjusted to lower the number required.

The biggest practical challenge with implementing these recommendations in practice is the availability of information on past $R_{CS}^{2}$ , given multinomial logistic regression CPMs are not (yet) very common. The proposed criteria will be most effective in achieving their aim when an accurate estimate of the $R_{CS}^{2}$ is available for both the multinomial model ( $R_{CS\_adj}^{2}$ ) and each distinct logistic regression model {k,r} ( $R_{CS\_adj,k,r}^{2}$ ). We have given advice on how to pre-specify these, but also want to urge researchers to report the relevant information when developing a multinomial logistic regression to enable this process. Currently, there is no way to estimate $R_{CS\_adj}^{2}$ for the multinomial model from metrics which are not pseudo- $R^{2}$ (for example there is no way to estimate it from the PDI^35^), meaning reporting $R_{CS\_adj}^{2}$ is very important. Estimates of $R_{CS\_adj,k,r}^{2}$ can be obtained from a variety of metrics from previously published ‘one-to-one’ distinct logistic regression models.^12,15^ However, when fitting a multinomial logistic regression, it is important to (at least) report the pairwise C-statistics using the conditional risk method^32^ when fitting a multinomial logistic regression model. This is an informative performance metric that should be reported anyway, and it will allow future researchers to estimate $R_{CS\_adj,k,r}^{2}$ (as was done in our worked example). In theory, the conditional risk method could also be used to report $R_{CS\_adj,k,r}^{2}$ directly, although we are not aware of any instances of people doing this is in the literature.

There are five important areas of future work. First, to establish a relationship between the PDI,^35^ a commonly reported statistic for discrimination of a multinomial logistic regression model, and the distribution of the linear predictors of the sub-models. This relationship has been established for the C-statistic and logistic regression^36–39^ allowing $R_{CS}^{2}$ to be estimated when only the C-statistic is available.^15^ Secondly, the simulation (Appendix S1) found that there was poor agreement between the heuristic shrinkage factors and the sub-model-specific shrinkage factors when covariate effects were large and sample sizes were small (Table S3). This resulted in not having the desired level of shrinkage in the developed models. This finding extends to binary logistic regression, but it is not clear whether similar results would be found for continuous or time-to-event outcomes. Given the proposed criterion (i) for every outcome type^11,12^ targets the heuristic shrinkage factor to be at the chosen threshold, it's important to establish in which scenarios where this may be a poor predictor of the sub-model specific shrinkage factors. Third, to extend the criteria of van Smeden et al.,^13^ to multinomial logistic regression. Their work acts as a fourth criterion,^14^ to target the mean absolute prediction error (MAPE) of a binary logistic regression model to be below a pre-specified threshold. This helps ensure precise predictions across the spectrum of predicted values. The formula is derived from a detailed simulation, in which a variety of binary logistic regression models are simulated and the MAPE assessed when the model is applied to new individuals from the target population. The first step to extending this criteria to multinomial logistic regression would be to define an extension of the MAPE for multinomial outcomes, which would then need to be followed by a similar simulation as the one used to derive the formula for binary logistic regression. The fourth is to develop sample size formula for the prediction of ordinal outcomes. The sample size formula proposed in this study are for a multinomial logistic regression, which can be fit to either nominal or ordinal outcomes. However if wanting to predict an ordinal outcome, an ordinal model could be fitted which would likely require a smaller sample size since they require less parameters to be estimated (for example if a proportional odds assumption is made). While the sample size criteria proposed in this paper would be valid for the prediction of an ordinal outcome using multinomial logistic regression, it is a conservative estimate. Future work could develop less conservative sample size criteria developed specifically for ordinal regression modelling techniques. In clinical trials for ordinal outcomes the proportional odds assumption has been shown to impact the required sample size,^40^ and this would be no different for CPMs. The fifth is to explore the idea of reducing the number of candidate predictor parameters in specific sub-models of the multinomial logistic regression as a way to reduce the required sample size, as discussed in the ‘Recommendations if required sample size is too high’ section.

These sample size criteria will be embedded into existing software (pmsampsize in R^41^ and Stata^42^) so they can be widely implemented in practice.

## Supplemental Material

sj-docx-1-smm-10.1177_09622802231151220 - Supplemental material for Minimum sample size for developing a multivariable prediction model using multinomial logistic regression Supplemental material, sj-docx-1-smm-10.1177_09622802231151220 for Minimum sample size for developing a multivariable prediction model using multinomial logistic regression by Alexander Pate, Richard D Riley, Gary S Collins, Maarten van Smeden, Ben Van Calster, Joie Ensor and Glen P Martin in Statistical Methods in Medical Research

## Appendix

**Nomenclature**
CPM: clinical prediction model
$E_{k}$: number of individuals with outcome category k
K: number of outcome categories
LR: likelihood ratio test statistic
$m_{k,r}$: number of individuals with outcome category *k* or *r* that is required to target the shrinkage factor $S_{DL,k,r}$ to be above some pre-defined threshold
$n_{k,r}$: total number of individuals in the whole cohort that is required to target the shrinkage factor $S_{DL,k,r}$ to be above some pre-defined threshold $\left(=m_{k,r}/p_{k,r}\right)$
$p_{k}$: proportion of individuals that have outcome category ∈{k} ,
$p_{k,r}$: proportion of individuals that have outcome category ∈{k,r}
Q: number of predictors parameters considered for inclusion prior to any variable selection
$R_{CS\_adj}^{2}$: optimism adjusted estimate of Cox-Snell's definition of explained variation for a binary logistic regression model
$R_{CS\_adj,k}^{2}$: $R_{CS\_adj}^{2}$ for distinct logistic regression model for sub-model *k*, undefined reference category
$R_{CS\_adj,k,r}^{2}$: $R_{CS\_adj}^{2}$ for distinct logistic regression model for sub-model *k* and reference category r
$R_{CS\_app}^{2}$: Apparent estimate of Cox-Snell's definition of explained variation for a binary logistic regression model
$R_{CS\_app,k}^{2}$: $R_{CS\_app}^{2}$ for distinct logistic regression model for sub-model *k*, undefined reference category
$R_{CS\_app,k,r}^{2}$: $R_{CS\_app}^{2}$ for distinct logistic regression model for sub-model *k* and reference category r
$R_{\mathrm{Nagelkerke}}^{2}$: Nagelkerke's definition of explained variation $\left(R^{2}\right)$ , $=R_{CS}^{2}/\max(R_{CS}^{2})$ , where $R_{CS}^{2}$ can be either $R_{CS\_adj}^{2}$ or $R_{CS\_app}^{2}$
S: global shrinkage factor for a binary logistic regression model
$S_{DL,k}$: shrinkage factor of distinct logistic regression model for sub-model *k*, undefined reference category
$S_{DL,k,r}$: shrinkage factor of distinct logistic regression model for sub-model *k* and reference category r
$S_{MN,k}$: sub-model specific shrinkage factor using the multinomial recalibration framework for sub-model *k*, undefined reference category
$S_{MN,k,r}$: sub-model specific shrinkage factor using the multinomial recalibration framework for sub-model *k* and reference category r
$S_{VH}$: heuristic shrinkage factor for a binary logistic regression model
$S_{VH\_MN}$: heuristic shrinkage factor for a multinomial logistic regression model
$\varphi_{k,r}$: outcome proportion in category *k* relative to the reference category *r* $\left(=E_{k}/E_{k}+E_{r}\right)$
