# Supplementary material for: Minimum sample size for developing a multivariable prediction model using multinomial logistic regression
Source: Stat Methods Med Res. 2023 Jan 19;32(3):555–71. doi: 10.1177/09622802231151220 (PMC10012398; doi:10.1177/09622802231151220)
Supplement: sj-docx-1-smm-10.1177_09622802231151220 - Supplemental material for Minimum sample size for developing a multivariable prediction model using multinomial logistic regression [file sj-docx-1-smm-10.1177_09622802231151220.docx]

Supplementary Material - Minimum Sample Size for Developing a Multivariable Prediction Model using Multinomial Logistic Regression

Alexander Pate, PhD, Richard D Riley, PhD, Gary S Collins, PhD, Maarten van Smeden, PhD, Ben Van Calster, PhD, Joie Ensor, PhD, Glen P Martin, PhD

Contents

[1. Full details of simulation study investigating the properties of the proposed alternative to criterion (i) 2](#_Toc111044441)

[2. Discussion of variability of the sub-model specific shrinkage factors 12](#_Toc111044454)

[3. Derivation of formula for $max(RCS\_app2)$for multinomial models 14](#_Toc111044455)

[4. Implications of estimating $RCS\_adj2$ through $RNagelkerke2=0.15$ on criterion (ii) 16](#_Toc111044456)

[5. Code for worked example in section 5 of main manuscript 17](#_Toc111044457)

[6. References 28](#_Toc111044458)

$max(R_{CS\_app}^{2})$ $R_{CS\_adj}^{2}$ $R_{Nagelkerke}^{2}=0.15$

# Full details of simulation study investigating the properties of the proposed alternative to criterion (i)

## Methods

Methods reported using the ADEMP structure.^1^ The simulation was carried out in R^2^ version 3.4.1 and RStudio^3^ using packages VGAM,^4^ foreach^5^ and doParallel.^6^ Reproducible code is provided at the referenced GitHub repository.^7^

### Simulation aims

A simulation study was carried out that evaluated the performance of the suggested ways to implement criterion (i) for multinomial logistic regression outlined in sections 3.2.1 and 3.2.2. Our primary aim was to investigate whether the sub-model specific shrinkage factors from the multinomial calibration framework were greater-than-or-equal to the pre-defined threshold used in the sample size calculations. Note that criterion (i) aims to keep the sub-model specific shrinkage factors above the pre-defined threshold *on average*, therefore we are interested in whether the mean and median of the sub-model specific shrinkage factors across all the simulations are above the threshold, rather than in every simulated dataset. Secondly, the simulation investigated whether the sub-model specific shrinkage factors in the multinomial logistic regression matched the distinct logistic shrinkage factors, to test the theory behind our proposed solution outlined in section 3.2.2.

For brevity, we only explore these aims for a single reference category. If our proposed sample size calculation approach is successful at keeping the multinomial sub-model specific shrinkage factors with the chosen reference category above the pre-specified threshold, then this would extend to repeating the process with the other reference categories. Our proposition in section 3.2.4 of repeating the process for each reference category, and taking the maximum sample size across all of them, will then be sufficient for targeting all the multinomial sub-model specific shrinkage factors to be above the pre-specified threshold.

As a brief overview, we simulated artificial development datasets for a variety of sample sizes, two of which met our proposed sample size criteria from section 3.2. We then fit multinomial logistic regression models and distinct binary logistic regression models to these datasets. Sub-model specific shrinkage factors ($S_{MN,k,r})$ and distinct logistic shrinkage factors $(S_{DL,k,r})$ were obtained using the validation process given in section 3.2.2, in a large simulated dataset representing the population of interest. This involved fitting the recalibration framework of van Hoorde et al.,^8^ and binary logistic recalibration techniques^9–12^. $S_{MN,k,r}$ and$S_{DL,k,r}$ were then compared to the targeted threshold of 0.9, and each other. Sample sizes using the proposed criteria were calculated based on ‘available information’ from models which we fitted to another artificial dataset, to mimic the process which would happen in practice, of obtaining estimates of $R_{CS}^{2}$ from previously available models.

### Data generating mechanisms and calculation of $N_{MN}$ and $N_{DL}$

Data were generated for a three-category outcome,$Y\in\{1,2,3\}$. Five independent predictor variables $X_{1}$, $X_{2}$, $X_{3}$, $X_{4}$, $X_{5}$ were generated by simulating $X_{i}\sim N\left( 0,1 \right), i \in\{1,2,3,4,5\}$, for N individuals (values of N described below). Ten different scenarios were considered by varying the coefficients of these predictor variables, with the aim of varying the outcome proportion in the different outcome categories. The model coefficients are denoted by $\beta_{0,2}$, $\beta_{1,2}$,$\beta_{2,2}$,$\beta_{3,2}$, $\beta_{4,2}$, $\beta_{5,2}$ and $\beta_{0,3}$, $\beta_{1,3}$,$\beta_{2,3}$,$\beta_{3,3}$, $\beta_{4,3}$, $\beta_{5,3}$, with values considered in the simulation scenarios given in Supplementary Table S1. For each individual, outcome data were simulated through a multinomial distribution with probabilities based on the following set of equations:^13^

$$P\left( Y=1 \right)=\frac{1}{1+\exp\left( \beta_{0,2}+\sum_{q=1}^{5} \beta_{q,2}X_{q} \right)+\exp\left( \beta_{0,3}+\sum_{q=1}^{5} \beta_{q,3}X_{q} \right)}$$

$$P\left( Y=2 \right)=\frac{\exp\left( \beta_{0,2}+\sum_{q=1}^{5} \beta_{q,2}X_{q} \right)}{1+\exp\left( \beta_{0,2}+\sum_{q=1}^{5} \beta_{q,2}X_{q} \right)+\exp\left( \beta_{0,3}+\sum_{q=1}^{5} \beta_{q,3}X_{q} \right)}$$

$$P\left( Y=3 \right)=\frac{\exp\left( \beta_{0,3}+\sum_{q=1}^{5} \beta_{q,3}X_{q} \right)}{1+\exp\left( \beta_{0,2}+\sum_{q=1}^{5} \beta_{q,2}X_{q} \right)+\exp\left( \beta_{0,3}+\sum_{q=1}^{5} \beta_{q,3}X_{q} \right)}$$

The values of $\beta$ and corresponding category outcome proportions in each scenario are provided in Supplementary Table S1. Scenarios 7 to 12 have covariate effects twice as big as scenarios 1 to 6. The intercepts of each outcome category model were selected so that simulation scenarios 1 and 7 represent a balanced outcome proportion situation, simulation scenarios 2 and 8 represent a ‘one lower’ outcome proportion situation, simulation scenarios 3 and 9 represent an ‘all different’ outcome proportion situation, simulation scenarios 4 and 10 represent a ‘one rare category’ situation, and simulation scenarios 5 and 11 represent a ‘one very rare category’ situation. The outcome proportion of the ‘very rare category’ in scenarios 6 and 12 (6%), was chosen to match the outcome proportion of borderline malignant (the rarest category), in the case study of ovarian cancer by de Jong et al.^14^ Without loss of generality, we chose $Y=1$ to always be the most prevalent category, and this was always chosen to be the reference category when fitting models and calibrating. This is often a reasonable approach to take in clinical prediction, when the covariate effects are not of direct interest.

We denote $N_{MN}$ to be the minimum sample size calculated by applying criterion (i) using the approach from section3.2.1, and $N_{DL}$ using the approach from section 3.2.2 based on distinct logistic regression models. For both we take category 1 to be the reference category. For each simulation scenario, we generated 1000 development datasets of size $N=100, 250, 500, 1000$, $N_{MN}$ and $N_{DL}$. For each scenario, a validation dataset of size $500,000$ was generated under the same data generating mechanisms as described above to represent a population, in which the required shrinkage of the models was assessed.

Calculation of $N_{MN}$ and $N_{DL}$

To calculate the minimum required sample sizes ($N_{MN}$ and $N_{DL}$) to satisfy criterion (i) for each scenario, we mimicked what would happen in practice by basing the sample size calculation off available information that would be available a priori. Specifically, for each scenario a multinomial logistic regression model and a set of distinct logistic regression models were fitted to a cohort of $500,000$ individuals. This dataset is different from the validation dataset. The $R_{CS\_adj}^{2}$ of these models was then assumed to be “publically available” and was utilised in the sample size calculations for each scenario, following the processes outlined in sections 3.2.1 and 3.2.2. A cohort size of $500,000$ was chosen to ensure the value of $R_{CS\_adj}^{2}$ used in the sample size calculation was accurate$.$ Both sample sizes were calculated with the intention targeting a shrinkage factor of $0.9$. The exact process for calculating these is detailed in the supplementary methods.

For each scenario, $N_{MN}$ was calculated as follows:

1. Generate a cohort of size $500,000$ using the aforementioned data generating mechanisms

2. Fit a multinomial model with X_1_ to X_5_ as predictors (equation (6)), and fit an intercept only multinomial model (null model) to this dataset.

3. Calculate the likelihood ratio as $LR=-2({loglikelihood}_{null} - {loglikelihood}_{full})$

4. Calculate $R_{CS\_app}^{2}=1-exp({LR}/{500000)}$

5. Calculate $S_{VH\_MN}=1+\frac{10}{500000*log(1-R_{CS\_app}^{2})}$

6. Calculate $R_{CS\_adj}^{2}=S_{VH\_MN}\times R_{CS\_app}^{2}$

7. Calculate $N_{MN}=\frac{10}{\left( 0.9-1 \right)*log(1-{R_{CS\_adj}^{2}}/{0.9})}$

Note, that $p$= 10, as although there are only 5 predictors, there are 10 predictor parameters to be calculated in the multinomial model. Step 7 could be replaced by using the *pmsampsize* package in R,^15^ using the value of $R_{adj}^{2}$ calculated from step 6.

For each scenario, $N_{DL}$ was calculated as follows:

1. Generate a cohort of size $500000$ using the aforementioned data generating mechanisms

2. Fit distinct logistic regression models with X_1_ to X_5_ as predictors (full models), and intercept only distinct logistic regression models (null models), to predict $log\left( \frac{P(Y=2)}{P(Y=1)} \right)$ and $log\left( \frac{P(Y=3)}{P(Y=1)} \right)$, on the subsets of the cohort which have the appropriate outcomes. These models match the sub-models from the multinomial framework.

3. Calculate the corresponding likelihood ratio from the models in step 2, as ${LR}_{k,1}=-2\left( {loglikelihood}_{null,k,1} - {loglikelihood}_{full,k,1} \right)$, for each model $k$.

Repeat the following steps 4 to 7 for both models ($k=1,2$) to calculate $n_{2,1}$ and $n_{3,1}$ from Box 1 (summary box):

4. Calculate $R_{CS\_app,k,1}^{2}=1-exp({{LR}_{k,1}}/{\sum_{i=1}^{500000} I(Y\in\left\{ 1,k \right\}))}$

5. Calculate $S_{VH\_DL,k,1}=1+\frac{5}{\sum_{i=1}^{500000} I(Y\in\left\{ 1,k \right\})*log(1-R_{CS\_app,k}^{2})}$

6. Calculate $R_{CS\_adj,k,1}^{2}=S_{VH\_DL,k,1}\times R_{CS\_app,k,1}^{2}$

7. Calculate $\omega_{k,i}=\frac{\sum_{i=1}^{500000} I(Y\in\left\{ 1,k \right\})}{500000}$

8. Calculate $m_{k,1}=\frac{5}{\left( 0.9-1 \right)*\log(1-{R_{CS\_adj,k,1}^{2}}/{0.9})}$

9. Calculate $n_{k,1}={m_{k,1}}/{\omega_{k,1}}$

8. Take $N_{DL}=max(n_{2,1},n_{3,1})$.

Note that $p$ = 5 in these calculations, as there are five predictors in each model, which is independent from the other model. In step 7, the sample size required to target the threshold in each distinct logistic regression model, is divided by the proportion of individuals from the initial cohort that would be used in this sub-model.

### Estimands

The Estimands of interest were:

$S_{MN,2,1}$ and $S_{MN,3,1}$: sub-model specific shrinkage factors. These values therefore represented the level of shrinkage required when the multinomial model was implemented in the population of interest.

$S_{DL,2,1}$ and $S_{DL,3,1}$: distinct logistic shrinkage factors

$S_{VH\_MN}$: heuristic shrinkage factor of the multinomial model

$S_{VH\_DL,2,1}$ and $S_{VH\_DL,3,1}$: heuristic shrinkage factors of the distinct logistic regression models

### Methods and models for comparison

A multinomial logistic regression and distinct logistic regression models with $X_{1}$ to $X_{5}$ as predictors were fitted to each development dataset. The sub-model specific shrinkage factors of the multinomial models ($S_{MN,2,1}$ and $S_{MN,3,1}$) were calculated by implementing the calibration framework of van Hoorde et al.^8^ in the validation cohort, with category 1 as the reference category. The distinct logistic shrinkage factors ($S_{DL,2,1}$ and $S_{DL,3,1}$), were calculated using binary logistic calibration techniques^9–12^ to calibrate the models in the validation cohort. This follows the process outlined in section 3.2.2. $S_{VH\_MN}$ was the heuristic shrinkage factor of the multinomial model, and $S_{VH\_DL,2,1}$ and $S_{VH\_DL,3,1}$ were the heuristic shrinkage factors of the distinct logistic regression models, calculated using equations (13) and (3) respectively.

The methods used to produce the estimands (described in this section) were not being compared in this simulation. Instead, we were comparing the value of these estimands when different sample sizes were used to generate the dataset. In particular, when $N=N_{MN}$ or $N_{DL}$.

### Performance measures and comparisons

We do not use standard performance measures given we are not comparing the ability of different methods to estimate an estimand of interest. The main performance measures are the mean and median of the sub-model specific shrinkage factors and the distinct logistic shrinkage factors. We compare these estimates with the targeted threshold, and each other, in three main ways listed here:

1) Comparison of the median and mean (across the 1000 simulations) of the sub-model specific shrinkage factors, $S_{MN,2,1}$ and $S_{MN,3,1}$, to the threshold 0.9. This assess the ultimate aim of sample size criteria (i) for the multinomial model, which is to target $S_{MN,2,1}$ and $S_{MN,3,1}$ to be at 0.9 on average.

2) Comparison of the sub-model specific shrinkage factors of the multinomial model ($S_{MN,2,1}$ and $S_{MN,3,1}$) and distinct logistic shrinkage factors ($S_{DL,21}$ and $S_{DL,3,1}$). The process for deriving $N_{DL}$ given in section 3.2.2 was based on a proposition that the sub-model specific shrinkage factors of the multinomial model will tend towards the distinct logistic shrinkage factors as $N\to\infty$. This comparison will assess this proposition.

3) Comparison of the distinct logistic shrinkage factors ($S_{DL,2,1}$ and $S_{DL,3,1}$), and the corresponding heuristic shrinkage factors ($S_{VH\_DL,2,1}$ and $S_{VH\_DL,3,1}$). Given that the sample size criterion are based on ensuring the heuristic shrinkage factors are close to the pre-specified value, but the ultimate aim is ensure the actual required shrinkage is close to the pre-specified value, it was important to evaluate the agreement between the two.

Note that the variance of the sub-model specific shrinkage factors and distinct logistic shrinkage factors are not relevant to our research question, because the proposed sample size criteria do not target anything about the variance of the shrinkage factors. However, it is of wider interest to researchers to know how variable the level of required shrinkage in practice is. We therefore present data on the variability of $S_{MN,2,1}$ and $S_{MN,3,1}$ in Appendix section 2.

## Simulation results and discussion

In this section we are interested in comparing the median/mean of the shrinkage factors, and whenever we refer to median/mean, this is the median/mean of the stated values (i.e. $S_{MN,2,1}$ and $S_{MN,3,1}$) across the 1000 simulations.

### Comparison of the median and mean of the sub-model specific shrinkage factors, $S_{MN,2,1}$ and $S_{MN,3,1}$, to the threshold 0.9.

Our results suggest that basing criterion (i) on distinct logistic regressions ($N=N_{DL}$) achieved the aim of ensuring that the median/mean of the sub-model specific shrinkage factors of the multinomial model were $\geq$ $0.9$. In contrast, and as expected, applying the sample size formula directly to the multinomial model ($N=N_{MN}$) only achieved this aim in some scenarios.

For example, in scenarios 1 to 3 the values of $N_{MN}$ and $N_{DL}$ were very similar, and when $N=N_{MN}$ or $N_{DL}$ the median of $S_{MN,2,1}$ and $S_{MN,3,1}$were both close to $0.9$ (Supplementary Table S2). However, in scenarios 4 and 5 we observed larger differences between $N_{MN}$ and $N_{DL}$. When $N=N_{MN}$, median $S_{MN,2,1}$ was notably larger than $0.9$, whereas median $S_{MN,3,1}$ was notably lower than 0.9. Importantly, this means the sample size criterion was not achieved in every sub-model. In contrast, when $N=N_{DL}$ the median $S_{MN,2,1}$ and $S_{MN,3,1}$ were both $\geq$ $0.9$, meaning the sample size criterion was achieved in every sub-model. Furthermore, the model with the smaller sub-model specific shrinkage factor ($S_{MN,3,1})$ was very close to $0.9$, indicating the number of individuals included was about as small as it could be to still ensure criterion (i) is met.

The targeted level of shrinkage ($S_{MN,2,1}$ and $S_{MN,3,1}$ both at or above $0.9$) was consistently not met for scenarios 7 to 12 (Supplementary Table S3) for $N=N_{MN}$ or $N_{DL}$. This finding appeared to be due to poor agreement between the distinct logistic shrinkage factors and the corresponding heuristic shrinkage factor in these scenarios, and is discussed in more detail in section 1.3.3.

### Comparison of the sub-model specific shrinkage factors of the multinomial model ($S_{MN,2,1}$ and $S_{MN,3,1}$) and shrinkage factors of the distinct logistic models ($S_{DL,2,1}$ and $S_{DL,3,1}$).

Supplementary Table S2 shows there was strong agreement between the sub-model specific shrinkage factors of the multinomial model ($S_{MN,2,1}$ and $S_{MN,3,1}$), and the distinct logistic shrinkage factors ($S_{DL,2,1}$ and $S_{DL,3,1}$), with closer agreement as $N$ increased (as expected, since the two are identical as $N\to\infty$). Importantly, for $N=N_{DL}$ there appeared to be good agreement between the two, meaning the asymptotic equivalence was holding when $N=N_{DL}$. This supports the reasoning behind targeting the shrinkage of distinct logistic regression models, when the underlying aim is to target the multinomial sub-model specific shrinkage factors.

### Comparison of the distinct logistic shrinkage factors ($S_{DL,2,1}$ and $S_{DL,3,1}$), and the corresponding heuristic shrinkage factors ($S_{VH\_DL,2,1}$ and $S_{VH\_DL,3,1}$).

For scenarios 1 to 6 there was good agreement between the medians of $S_{DL,2,1}$ and $S_{DL,3,1}$ with the medians of $S_{VH\_DL,2,1}$ and $S_{VH\_DL,3,1}$, with no consistent pattern in the direction of the disagreement (Supplementary Table S2). There were slightly bigger differences in the mean, which is likely because $S_{VH\_DL,2,1}$ and $S_{VH\_DL,3,1}$ are bounded at 1, resulting in a skewed distribution, which is not the case for $S_{DL,2,1}$ and $S_{DL,3,1}$.

On the contrary, there was relatively poor agreement in the average $S_{DL,2,1}$ and $S_{DL,3,1}$ with the average $S_{VH\_DL,2,1}$ and $S_{VH\_DL,3,1}$ in scenarios 7 to 12 (Supplementary Table S3). Specifically, the medians and means of $S_{VH\_DL,2,1}$ and $S_{VH\_DL,3,1}$ were consistently higher than those of $S_{DL,2,1}$ and $S_{DL,3,1}$. These results suggest that our proposed criterion successfully ensured the median/mean value of both heuristic shrinkage factors was $\geq0.9$, but it did not ensure the median/mean value of the shrinkage required in the validation cohort was $\geq0.9$. This is an important because it is not unique to multinomial models. The major difference between scenarios 1 to 6 (where there was good agreement) and 7 to 12 (where $S_{VH\_DL,k,r}$ was consistently higher than $S_{DL,k,r}$) was that the covariate effect sizes were doubled in the latter. This in turn means the required sample sizes for scenarios 7 to 12 were much smaller than their counterparts in scenarios 1 to 6, as larger covariate effects require lower sample sizes to estimate. This simulation indicates that with these larger effect sizes and smaller sample sizes, the heuristic shrinkage factor no longer estimates the shrinkage factor upon validation unbiasedly. It may therefore not be enough to target the average heuristic shrinkage factor to be at $0.9$ in scenarios where covariate effects are expected a priori to be large. The mechanism behind this finding is unclear, and further work is required to understand this observation.

## Overall conclusions

1) We suggest basing the sample size for criterion (i) on the shrinkage of distinct logistic regression models (sections 3.2.2 - 3.2.4). In our simulations, $N_{DL}$ ensured the average value of all the sub-model specific shrinkage factors was $\geq$ $0.9$, as targeted. We advise against basing the sample size on the overall shrinkage of the multinomial model (section 3.2.1). In our simulations, ${N=N}_{MN}$ did not always result in the desired level of shrinkage in all sub-models. There were no scenarios that we considered where ${N=N}_{MN}$ outperformed ${N=N}_{DL}$ with regards to this aim of the sample size criterion.

2) There was good agreement between sub-model specific shrinkage factors ($S_{MN,2,1}$ and $S_{MN,3,1}$), and the distinct logistic shrinkage factors ($S_{DL,2,1}$ and $S_{DL,3,1}$), supporting the claim that these are equivalent as $N\to\infty$.

3) There was a poor agreement between the heuristic shrinkage factors and the distinct logistic shrinkage factors at model validation in scenarios 7 – 12 (increased covariate effect sizes), which resulted in sub-model specific shrinkage factors that were below the targeted threshold. This will need to be explored in future work and is relevant to binary logistic regression too.

## Tables

Supplementary Table S1: Beta coefficients and resulting outcome proportions


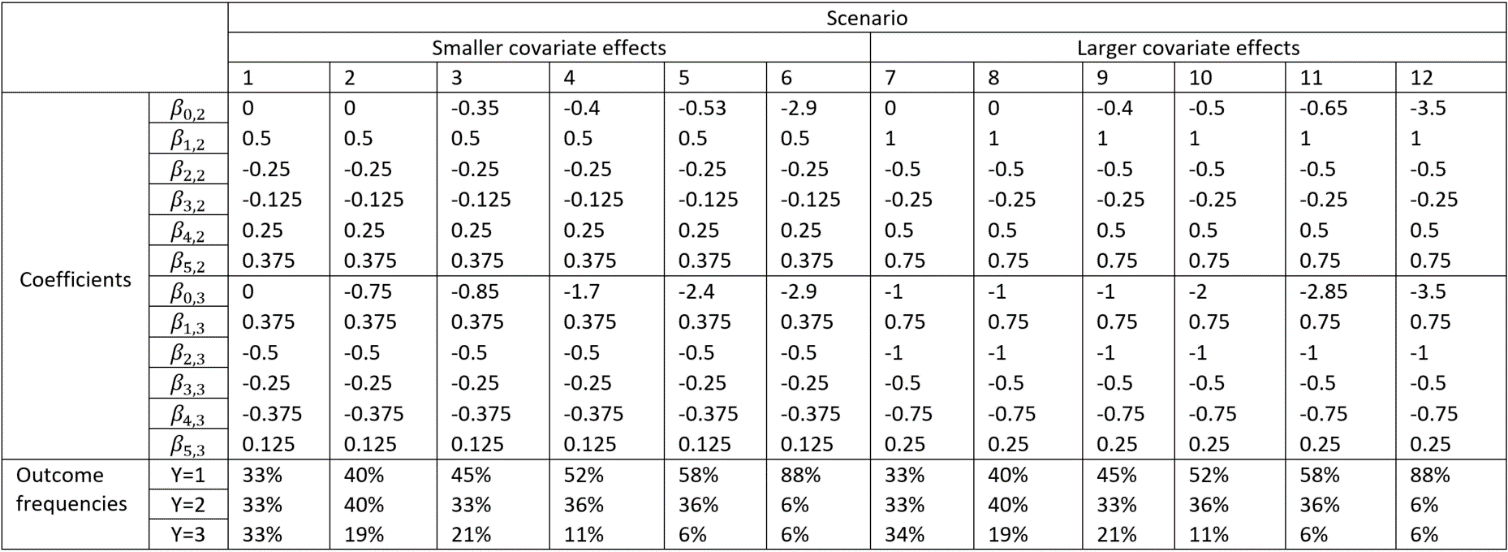


Supplementary Table S2: Median (mean) sub-model specific shrinkage factors and distinct logistic shrinkage factors, and heuristic shrinkage factors from multinomial and distinct logistic regression models, scenarios 7 to 12

| Scenario 1 (all same) | | | | | | | |
| --- | --- | --- | --- | --- | --- | --- | --- |
|  | Multinomial | | | Distinct logistic | | | |
| N | S_MN,2,1_ | S_MN,3,1_ | S_VH_MN_ | S_DL,2,1_ | S_DL,3,1_ | S_VH_DL,2,1_ | S_VH_DL,3,1_ |
| 250 | 0.783 (0.807) | 0.806 (0.828) | 0.802 (0.791) | 0.779 (0.806) | 0.785 (0.821) | 0.785 (0.759) | 0.811 (0.784) |
| 500 | 0.886 (0.904) | 0.892 (0.905) | 0.891 (0.888) | 0.884 (0.904) | 0.887 (0.904) | 0.882 (0.874) | 0.895 (0.889) |
| 1000 | 0.943 (0.948) | 0.943 (0.95) | 0.942 (0.942) | 0.933 (0.944) | 0.937 (0.95) | 0.938 (0.936) | 0.945 (0.944) |
| N_MN_ (541) | 0.893 (0.905) | 0.899 (0.912) | 0.899 (0.896) | 0.879 (0.899) | 0.899 (0.908) | 0.892 (0.884) | 0.902 (0.898) |
| N_DL_ (576) | 0.906 (0.92) | 0.902 (0.919) | 0.903 (0.901) | 0.903 (0.916) | 0.898 (0.918) | 0.895 (0.889) | 0.909 (0.903) |
| Scenario 2 (one lower) | | | | | | | |
|  | Multinomial | | | Distinct logistic | | | |
| N | S_MN,2,1_ | S_MN,3,1_ | S_VH_MN_ | S_DL,2,1_ | S_DL,3,1_ | S_VH_DL,2,1_ | S_VH_DL,3,1_ |
| 250 | 0.811 (0.829) | 0.771 (0.8) | 0.793 (0.78) | 0.814 (0.841) | 0.763 (0.794) | 0.811 (0.793) | 0.768 (0.736) |
| 500 | 0.908 (0.92) | 0.887 (0.899) | 0.884 (0.881) | 0.91 (0.929) | 0.88 (0.894) | 0.899 (0.893) | 0.871 (0.864) |
| 1000 | 0.951 (0.957) | 0.934 (0.946) | 0.94 (0.939) | 0.953 (0.963) | 0.931 (0.945) | 0.948 (0.946) | 0.934 (0.931) |
| N_MN_ (569) | 0.914 (0.921) | 0.891 (0.909) | 0.899 (0.896) | 0.917 (0.931) | 0.883 (0.908) | 0.911 (0.907) | 0.889 (0.879) |
| N_DL_ (628) | 0.926 (0.936) | 0.908 (0.923) | 0.906 (0.904) | 0.935 (0.944) | 0.909 (0.919) | 0.918 (0.915) | 0.894 (0.889) |
| Scenario 3 (all different) | | | | | | | |
|  | Multinomial | | | Distinct logistic | | | |
| N | S_MN,2,1_ | S_MN,3,1_ | S_VH_MN_ | S_DL,2,1_ | S_DL,3,1_ | S_VH_DL,2,1_ | S_VH_DL,3,1_ |
| 250 | 0.801 (0.826) | 0.772 (0.802) | 0.795 (0.781) | 0.809 (0.834) | 0.767 (0.8) | 0.808 (0.787) | 0.787 (0.756) |
| 500 | 0.899 (0.912) | 0.887 (0.901) | 0.884 (0.881) | 0.911 (0.921) | 0.879 (0.896) | 0.894 (0.889) | 0.88 (0.872) |
| 1000 | 0.939 (0.953) | 0.941 (0.948) | 0.939 (0.939) | 0.946 (0.959) | 0.936 (0.942) | 0.946 (0.944) | 0.937 (0.936) |
| N_MN_ (566) | 0.921 (0.925) | 0.89 (0.906) | 0.897 (0.894) | 0.919 (0.929) | 0.882 (0.897) | 0.906 (0.902) | 0.895 (0.888) |
| N_DL_ (582) | 0.911 (0.93) | 0.896 (0.909) | 0.9 (0.897) | 0.919 (0.937) | 0.887 (0.902) | 0.908 (0.903) | 0.898 (0.891) |
| Scenario 4 (one rare category) | | | | | | | |
|  | Multinomial | | | Distinct logistic | | | |
| N | S_MN,2,1_ | S_MN,3,1_ | S_VH_MN_ | S_DL,2,1_ | S_DL,3,1_ | S_VH_DL,2,1_ | S_VH_DL,3,1_ |
| 250 | 0.811 (0.837) | 0.699 (0.726) | 0.773 (0.76) | 0.825 (0.853) | 0.679 (0.713) | 0.827 (0.808) | 0.699 (0.638) |
| 500 | 0.899 (0.914) | 0.83 (0.851) | 0.873 (0.869) | 0.91 (0.925) | 0.83 (0.845) | 0.907 (0.901) | 0.825 (0.809) |
| 1000 | 0.952 (0.958) | 0.916 (0.931) | 0.932 (0.931) | 0.957 (0.963) | 0.909 (0.925) | 0.951 (0.95) | 0.906 (0.899) |
| N_MN_ (648) | 0.933 (0.943) | 0.865 (0.885) | 0.899 (0.896) | 0.939 (0.952) | 0.858 (0.883) | 0.925 (0.921) | 0.862 (0.85) |
| N_DL_ (901) | 0.936 (0.95) | 0.888 (0.909) | 0.927 (0.925) | 0.943 (0.958) | 0.886 (0.905) | 0.946 (0.945) | 0.9 (0.893) |
| Scenario 5 (one very rare category) | | | | | | | |
|  | Multinomial | | | Distinct logistic | | | |
| N | S_MN,2,1_ | S_MN,3,1_ | S_VH_MN_ | S_DL,2,1_ | S_DL,3,1_ | S_VH_DL,2,1_ | S_VH_DL,3,1_ |
| 250 | 0.8 (0.829) | 0.594 (0.622) | 0.76 (0.743) | 0.82 (0.844) | 0.583 (0.612) | 0.833 (0.815) | 0.598 (0.498) |
| 500 | 0.909 (0.915) | 0.772 (0.799) | 0.861 (0.856) | 0.92 (0.925) | 0.757 (0.793) | 0.906 (0.903) | 0.743 (0.695) |
| 1000 | 0.948 (0.955) | 0.878 (0.901) | 0.926 (0.925) | 0.952 (0.96) | 0.873 (0.897) | 0.952 (0.951) | 0.859 (0.845) |
| N_MN_ (706) | 0.93 (0.943) | 0.846 (0.868) | 0.898 (0.895) | 0.94 (0.952) | 0.843 (0.867) | 0.933 (0.93) | 0.808 (0.78) |
| N_DL_ (1458) | 0.967 (0.97) | 0.913 (0.931) | 0.948 (0.948) | 0.971 (0.974) | 0.918 (0.93) | 0.967 (0.966) | 0.899 (0.893) |
| Scenario 6 (two very rare categories) | | | | | | | |
|  | Multinomial | | | Distinct logistic | | | |
| N | S_MN,2,1_ | S_MN,3,1_ | S_VH_MN_ | S_DL,2,1_ | S_DL,3,1_ | S_VH_DL,2,1_ | S_VH_DL,3,1_ |
| 250 | 0.58 (0.61) | 0.585 (0.609) | 0.582 (0.529) | 0.589 (0.618) | 0.592 (0.617) | 0.537 (0.356) | 0.602 (0.489) |
| 500 | 0.764 (0.791) | 0.744 (0.78) | 0.738 (0.719) | 0.77 (0.798) | 0.752 (0.788) | 0.715 (0.66) | 0.762 (0.71) |
| 1000 | 0.861 (0.889) | 0.857 (0.881) | 0.855 (0.847) | 0.866 (0.893) | 0.86 (0.885) | 0.846 (0.831) | 0.864 (0.85) |
| N_MN_ (1558) | 0.924 (0.94) | 0.902 (0.92) | 0.9 (0.898) | 0.924 (0.943) | 0.909 (0.925) | 0.895 (0.888) | 0.909 (0.903) |
| N_DL_ (1616) | 0.927 (0.943) | 0.911 (0.924) | 0.902 (0.9) | 0.931 (0.946) | 0.917 (0.928) | 0.897 (0.891) | 0.91 (0.905) |

S_MN,k,r_, multinomial sub-model specific shrinkage factors; S_VH_MN_, heuristic shrinkage factor of multinomial model; S_DL,k,r_, distinct logistic shrinkage factors; S_VH_DL,k,r_, heuristic shrinkage factor of distinct logistic models.

Supplementary Table S3: Median (mean) sub-model specific shrinkage factors and distinct logistic shrinkage factors, and heuristic shrinkage factors from multinomial and distinct logistic regression models, scenarios 7 to 12

| Scenario 7 (all same) | | | | | | | |
| --- | --- | --- | --- | --- | --- | --- | --- |
|  | Multinomial | | | Distinct logistic | | | |
| N | S_MN,2,1_ | S_MN,3,1_ | S_VH_MN_ | S_DL,2,1_ | S_DL,3,1_ | S_VH_DL,2,1_ | S_VH_DL,3,1_ |
| 250 | 0.894 (0.904) | 0.898 (0.906) | 0.924 (0.923) | 0.879 (0.891) | 0.883 (0.894) | 0.918 (0.914) | 0.925 (0.922) |
| 500 | 0.942 (0.947) | 0.936 (0.944) | 0.961 (0.961) | 0.934 (0.939) | 0.923 (0.932) | 0.957 (0.956) | 0.962 (0.961) |
| 1000 | 0.97 (0.973) | 0.964 (0.967) | 0.98 (0.98) | 0.958 (0.965) | 0.958 (0.963) | 0.978 (0.978) | 0.98 (0.98) |
| N_MN_ (177) | 0.846 (0.862) | 0.849 (0.861) | 0.897 (0.895) | 0.831 (0.844) | 0.826 (0.841) | 0.887 (0.882) | 0.899 (0.894) |
| N_DL_ (196) | 0.866 (0.886) | 0.874 (0.884) | 0.906 (0.902) | 0.856 (0.875) | 0.846 (0.867) | 0.895 (0.889) | 0.906 (0.902) |
| Scenario 8 (one lower) | | | | | | | |
|  | Multinomial | | | Distinct logistic | | | |
| N | S_MN,2,1_ | S_MN,3,1_ | S_VH_MN_ | S_DL,2,1_ | S_DL,3,1_ | S_VH_DL,2,1_ | S_VH_DL,3,1_ |
| 250 | 0.895 (0.904) | 0.874 (0.891) | 0.92 (0.919) | 0.897 (0.905) | 0.85 (0.865) | 0.93 (0.928) | 0.909 (0.905) |
| 500 | 0.941 (0.95) | 0.932 (0.94) | 0.959 (0.958) | 0.945 (0.948) | 0.917 (0.925) | 0.964 (0.963) | 0.953 (0.952) |
| 1000 | 0.968 (0.971) | 0.962 (0.965) | 0.979 (0.979) | 0.972 (0.97) | 0.96 (0.961) | 0.982 (0.982) | 0.976 (0.975) |
| N_MN_ (189) | 0.866 (0.88) | 0.836 (0.851) | 0.897 (0.895) | 0.862 (0.879) | 0.812 (0.832) | 0.909 (0.905) | 0.884 (0.876) |
| N_DL_ (219) | 0.883 (0.892) | 0.874 (0.881) | 0.91 (0.908) | 0.885 (0.893) | 0.84 (0.858) | 0.921 (0.918) | 0.896 (0.891) |
| Scenario 9 (all different) | | | | | | | |
|  | Multinomial | | | Distinct logistic | | | |
| N | S_MN,2,1_ | S_MN,3,1_ | S_VH_MN_ | S_DL,2,1_ | S_DL,3,1_ | S_VH_DL,2,1_ | S_VH_DL,3,1_ |
| 250 | 0.895 (0.906) | 0.888 (0.896) | 0.921 (0.92) | 0.888 (0.902) | 0.861 (0.878) | 0.927 (0.925) | 0.918 (0.914) |
| 500 | 0.952 (0.957) | 0.943 (0.953) | 0.959 (0.958) | 0.953 (0.957) | 0.934 (0.944) | 0.962 (0.961) | 0.957 (0.956) |
| 1000 | 0.973 (0.977) | 0.968 (0.97) | 0.979 (0.979) | 0.976 (0.979) | 0.962 (0.965) | 0.981 (0.981) | 0.978 (0.978) |
| N_MN_ (186) | 0.86 (0.873) | 0.849 (0.863) | 0.898 (0.895) | 0.861 (0.87) | 0.831 (0.846) | 0.905 (0.9) | 0.893 (0.886) |
| N_DL_ (198) | 0.86 (0.877) | 0.856 (0.873) | 0.904 (0.901) | 0.857 (0.873) | 0.839 (0.856) | 0.911 (0.906) | 0.898 (0.892) |
| Scenario 10 (one rare category) | | | | | | | |
|  | Multinomial | | | Distinct logistic | | | |
| N | S_MN,2,1_ | S_MN,3,1_ | S_VH_MN_ | S_DL,2,1_ | S_DL,3,1_ | S_VH_DL,2,1_ | S_VH_DL,3,1_ |
| 250 | 0.899 (0.904) | 0.855 (0.874) | 0.913 (0.911) | 0.904 (0.912) | 0.834 (0.849) | 0.934 (0.932) | 0.884 (0.875) |
| 500 | 0.949 (0.953) | 0.924 (0.935) | 0.954 (0.954) | 0.953 (0.956) | 0.924 (0.933) | 0.966 (0.965) | 0.938 (0.936) |
| 1000 | 0.973 (0.976) | 0.958 (0.965) | 0.977 (0.977) | 0.978 (0.98) | 0.954 (0.959) | 0.983 (0.982) | 0.968 (0.968) |
| N_MN_ (209) | 0.876 (0.886) | 0.824 (0.842) | 0.898 (0.895) | 0.883 (0.892) | 0.792 (0.819) | 0.922 (0.919) | 0.864 (0.851) |
| N_DL_ (289) | 0.908 (0.922) | 0.865 (0.886) | 0.924 (0.922) | 0.913 (0.926) | 0.859 (0.87) | 0.942 (0.94) | 0.897 (0.89) |
| Scenario 11 (one very rare category) | | | | | | | |
|  | Multinomial | | | Distinct logistic | | | |
| N | S_MN,2,1_ | S_MN,3,1_ | S_VH_MN_ | S_DL,2,1_ | S_DL,3,1_ | S_VH_DL,2,1_ | S_VH_DL,3,1_ |
| 250 | 0.897 (0.907) | 0.807 (0.826) | 0.903 (0.901) | 0.908 (0.918) | 0.773 (0.793) | 0.935 (0.933) | 0.827 (0.805) |
| 500 | 0.952 (0.957) | 0.903 (0.916) | 0.949 (0.949) | 0.957 (0.962) | 0.879 (0.895) | 0.966 (0.966) | 0.907 (0.901) |
| 1000 | 0.977 (0.978) | 0.955 (0.964) | 0.974 (0.974) | 0.976 (0.98) | 0.947 (0.953) | 0.983 (0.983) | 0.951 (0.95) |
| N_MN_ (233) | 0.888 (0.902) | 0.791 (0.805) | 0.899 (0.896) | 0.902 (0.914) | 0.753 (0.772) | 0.931 (0.929) | 0.82 (0.794) |
| N_DL_ (455) | 0.939 (0.944) | 0.887 (0.9) | 0.945 (0.944) | 0.942 (0.949) | 0.866 (0.877) | 0.964 (0.963) | 0.901 (0.893) |
| Scenario 12 (two very rare categories) | | | | | | | |
|  | Multinomial | | | Distinct logistic | | | |
| N | S_MN,2,1_ | S_MN,3,1_ | S_VH_MN_ | S_DL,2,1_ | S_DL,3,1_ | S_VH_DL,2,1_ | S_VH_DL,3,1_ |
| 250 | 0.779 (0.789) | 0.787 (0.806) | 0.826 (0.816) | 0.775 (0.79) | 0.787 (0.811) | 0.813 (0.782) | 0.842 (0.817) |
| 500 | 0.871 (0.888) | 0.881 (0.896) | 0.905 (0.902) | 0.878 (0.888) | 0.884 (0.899) | 0.899 (0.891) | 0.913 (0.908) |
| 1000 | 0.926 (0.933) | 0.939 (0.942) | 0.951 (0.95) | 0.921 (0.933) | 0.941 (0.943) | 0.947 (0.945) | 0.955 (0.954) |
| N_MN_ (470) | 0.862 (0.873) | 0.876 (0.89) | 0.9 (0.896) | 0.859 (0.873) | 0.878 (0.891) | 0.891 (0.883) | 0.908 (0.902) |
| N_DL_ (505) | 0.875 (0.89) | 0.892 (0.903) | 0.906 (0.903) | 0.874 (0.891) | 0.893 (0.902) | 0.899 (0.891) | 0.915 (0.909) |

S_MN,k,r_, multinomial sub-model specific shrinkage factors; S_VH_MN_, heuristic shrinkage factor of multinomial model; S_DL,k,r_, distinct logistic shrinkage factors; S_VH_DL,k,r_, heuristic shrinkage factor of distinct logistic models.

# Discussion of variability of the sub-model specific shrinkage factors

Supplementary Table S4 provides the full distribution of $S_{MN,2,1}$ and $S_{MN,3,1}$. We see that there is a reasonable amount of variability in the sub-model specific shrinkage factors across the 1000 simulations. When ${N=N}_{DL}$, the 25^th^ percentile for the smaller sub-model specific shrinkage factor was approximately $0.825$, meaning in our simulation 25% of models meeting the sample size criteria, the shrinkage factor of that sub-model was $\leq0.825$. This is an important result given that in practice you only get to recruit one cohort of individuals and build one model, and for many shrinkage methods the estimated shrinkage is inversely proportional to the actual amount of shrinkage required.^16,17^ While important, it was also to be expected. The sample size criteria target the average shrinkage factor to be at a pre-specified threshold, in which they are successful. Our findings agree with previous work which reported on the variability in model performance when the sample size criteria are met.^18^ We report on this to re-iterate to model users and developers that meeting the sample size criteria will not ensure the required shrinkage of your model is above $0.9$. Shrinkage techniques are therefore still advised when sample size criteria are met,^18^ however even this is not a full-proof solution to the issue of overfitting.^16,19^ If it is possible to use a sample size corresponding a higher threshold than $0.9$ this is encouraged, as it will reduce both the average and variability in the amount of shrinkage required. If feasible in the data collection process, an adaptive sample size approach^20^ could be taken where the level of overfitting in the developed model is monitored, with recruitment/data collection stopping after the level of overfitting reaches $0.9$. This means the desired level of shrinkage should be achieved in the model of interest, which may not be the case when calculating a fixed sample size prior to model development.

Supplementary Table S4: 2.5^th^, 25^th^, 50^th^, 75^th^, 97.5^th^ percentile of sub-model specific shrinkage factors, S_MN,2,1_ and S_MN,3,1_, across the 1000 simulations, scenarios 1 to 6

| Scenario 1 (all same) | | | | | | | | | | |
| --- | --- | --- | --- | --- | --- | --- | --- | --- | --- | --- |
|  | S_MN,2,1_ | | | | | S_MN,3,1_ | | | | |
| N | 2.5^th^ | 25^th^ | 50^th^ | 75^th^ | 97.5^th^ | 2.5^th^ | 25^th^ | 50^th^ | 75^th^ | 97.5^th^ |
| 250 | 0.549 | 0.693 | 0.783 | 0.907 | 1.193 | 0.571 | 0.711 | 0.806 | 0.914 | 1.230 |
| 500 | 0.684 | 0.815 | 0.886 | 0.982 | 1.211 | 0.695 | 0.814 | 0.892 | 0.978 | 1.199 |
| 1000 | 0.780 | 0.876 | 0.943 | 1.010 | 1.139 | 0.788 | 0.880 | 0.943 | 1.015 | 1.154 |
| N_MN_ (541) | 0.687 | 0.810 | 0.893 | 0.980 | 1.210 | 0.702 | 0.830 | 0.899 | 0.987 | 1.179 |
| N_DL_ (576) | 0.711 | 0.836 | 0.906 | 0.989 | 1.209 | 0.720 | 0.827 | 0.902 | 0.992 | 1.193 |
| Scenario 2 (one lower) | | | | | | | | | | |
|  | S_MN,2,1_ | | | | | S_MN,3,1_ | | | | |
| N | 2.5^th^ | 25^th^ | 50^th^ | 75^th^ | 97.5^th^ | 2.5^th^ | 25^th^ | 50^th^ | 75^th^ | 97.5^th^ |
| 250 | 0.567 | 0.709 | 0.811 | 0.916 | 1.215 | 0.529 | 0.675 | 0.771 | 0.893 | 1.218 |
| 500 | 0.704 | 0.825 | 0.908 | 0.990 | 1.219 | 0.661 | 0.798 | 0.887 | 0.981 | 1.217 |
| 1000 | 0.782 | 0.889 | 0.951 | 1.016 | 1.189 | 0.772 | 0.873 | 0.934 | 1.012 | 1.172 |
| N_MN_ (569) | 0.706 | 0.827 | 0.914 | 0.997 | 1.198 | 0.688 | 0.811 | 0.891 | 0.991 | 1.271 |
| N_DL_ (628) | 0.735 | 0.846 | 0.926 | 1.010 | 1.184 | 0.698 | 0.834 | 0.908 | 1.002 | 1.253 |
| Scenario 3 (all different) | | | | | | | | | | |
|  | S_MN,2,1_ | | | | | S_MN,3,1_ | | | | |
| N | 2.5^th^ | 25^th^ | 50^th^ | 75^th^ | 97.5^th^ | 2.5^th^ | 25^th^ | 50^th^ | 75^th^ | 97.5^th^ |
| 250 | 0.567 | 0.708 | 0.801 | 0.915 | 1.228 | 0.525 | 0.671 | 0.772 | 0.898 | 1.231 |
| 500 | 0.681 | 0.815 | 0.899 | 0.993 | 1.211 | 0.677 | 0.802 | 0.887 | 0.980 | 1.220 |
| 1000 | 0.784 | 0.880 | 0.939 | 1.015 | 1.196 | 0.771 | 0.872 | 0.941 | 1.009 | 1.175 |
| N_MN_ (566) | 0.705 | 0.832 | 0.921 | 1.003 | 1.203 | 0.696 | 0.814 | 0.890 | 0.988 | 1.187 |
| N_DL_ (582) | 0.714 | 0.838 | 0.911 | 1.007 | 1.253 | 0.684 | 0.817 | 0.896 | 0.985 | 1.214 |
| Scenario 4 (one rare category) | | | | | | | | | | |
|  | S_MN,2,1_ | | | | | S_MN,3,1_ | | | | |
| N | 2.5^th^ | 25^th^ | 50^th^ | 75^th^ | 97.5^th^ | 2.5^th^ | 25^th^ | 50^th^ | 75^th^ | 97.5^th^ |
| 250 | 0.586 | 0.720 | 0.811 | 0.921 | 1.254 | 0.423 | 0.586 | 0.699 | 0.822 | 1.270 |
| 500 | 0.696 | 0.824 | 0.899 | 0.991 | 1.214 | 0.592 | 0.729 | 0.830 | 0.949 | 1.243 |
| 1000 | 0.789 | 0.889 | 0.952 | 1.017 | 1.165 | 0.713 | 0.837 | 0.916 | 1.005 | 1.227 |
| N_MN_ (648) | 0.735 | 0.862 | 0.933 | 1.015 | 1.218 | 0.646 | 0.777 | 0.865 | 0.963 | 1.260 |
| N_DL_ (901) | 0.780 | 0.880 | 0.936 | 1.011 | 1.172 | 0.689 | 0.812 | 0.888 | 0.984 | 1.220 |
| Scenario 5 (one very rare category) | | | | | | | | | | |
|  | S_MN,2,1_ | | | | | S_MN,3,1_ | | | | |
| N | 2.5^th^ | 25^th^ | 50^th^ | 75^th^ | 97.5^th^ | 2.5^th^ | 25^th^ | 50^th^ | 75^th^ | 97.5^th^ |
| 250 | 0.569 | 0.708 | 0.800 | 0.917 | 1.210 | 0.260 | 0.478 | 0.594 | 0.737 | 1.118 |
| 500 | 0.681 | 0.824 | 0.909 | 0.996 | 1.194 | 0.501 | 0.660 | 0.772 | 0.897 | 1.290 |
| 1000 | 0.782 | 0.889 | 0.948 | 1.013 | 1.173 | 0.656 | 0.794 | 0.878 | 0.986 | 1.259 |
| N_MN_ (706) | 0.751 | 0.856 | 0.930 | 1.019 | 1.203 | 0.586 | 0.744 | 0.846 | 0.962 | 1.291 |
| N_DL_ (1458) | 0.827 | 0.915 | 0.967 | 1.019 | 1.137 | 0.717 | 0.842 | 0.913 | 1.008 | 1.214 |
| Scenario 6 (two very rare categories) | | | | | | | | | | |
|  | S_MN,2,1_ | | | | | S_MN,3,1_ | | | | |
| N | 2.5^th^ | 25^th^ | 50^th^ | 75^th^ | 97.5^th^ | 2.5^th^ | 25^th^ | 50^th^ | 75^th^ | 97.5^th^ |
| 250 | 0.201 | 0.454 | 0.580 | 0.732 | 1.199 | 0.273 | 0.476 | 0.585 | 0.708 | 1.109 |
| 500 | 0.477 | 0.652 | 0.764 | 0.901 | 1.254 | 0.480 | 0.654 | 0.744 | 0.876 | 1.237 |
| 1000 | 0.636 | 0.768 | 0.861 | 0.982 | 1.282 | 0.635 | 0.775 | 0.857 | 0.960 | 1.313 |
| N_MN_ (1558) | 0.719 | 0.835 | 0.924 | 1.024 | 1.277 | 0.694 | 0.824 | 0.902 | 0.995 | 1.233 |
| N_DL_ (1616) | 0.725 | 0.839 | 0.927 | 1.020 | 1.285 | 0.694 | 0.834 | 0.911 | 0.998 | 1.219 |

S_MN,k,r_, multinomial sub-model specific shrinkage factors.

# Derivation of formula for $\mathbf{max}\boldsymbol{(}\boldsymbol{R}_{\boldsymbol{CS\_app}}^{\boldsymbol{2}}\boldsymbol{)}$for multinomial models

We provide this proof as we are not aware of seeing this results in the literature.

Starting from equation (25):

$lnL_{null}=\sum_{k=1}^{K} E_{k}ln\left( \frac{E_{k}}{n} \right)$,

And equation (16):

$\max\left( R_{CS\_app}^{2} \right)=1-\exp\left( \frac{2\ln L_{null}}{n} \right)$,

We get that:

$$\max\left( R_{CS\_app}^{2} \right)=1-\exp\left( \frac{2*\sum_{k=1}^{K} E_{k}\ln\left( \frac{E_{k}}{n} \right)}{n} \right)$$

$$=1-\exp\left[ \sum_{k=1}^{K} \frac{2*E_{k}\ln\left( \frac{E_{k}}{n} \right)}{n} \right]$$

$$=1-\prod_{k=1}^{K} \exp\left[ \frac{2*E_{k}\ln\left( \frac{E_{k}}{n} \right)}{n} \right]$$

$$=1-\prod_{k=1}^{K} \exp\left[ \ln\left( \left( \frac{E_{k}}{n} \right)^{\frac{2*E_{k}}{n}} \right) \right]$$

$$=1-\prod_{k=1}^{K} \left( \frac{E_{k}}{n} \right)^{\frac{2*E_{k}}{n}}$$

$$=1-\left( \prod_{k=1}^{K} \left( \frac{E_{k}}{n} \right)^{\frac{E_{k}}{n}} \right)^{2}$$

$$=1-\left( \prod_{k=1}^{K} \left( p_{k} \right)^{p_{k}} \right)^{2}$$

where ${{p_{k}=E}_{k}}/n$ is the is the observed frequency of category $k$, as defined in section 3.4 of the main paper.

Now, for binary logistic regression $\max\left( R_{CS\_app}^{2} \right)$ has a maximum of 0.75, when the observed outcome frequency is = 0.5.

For multinomial logistic regression, $\max\left( R_{CS\_app}^{2} \right)$ occurs when all the observed outcome frequencies are equal, i.e. $p_{k}=1/K: k=1,\ldots,K$. This results in a maximum (across all possible outcome frequencies) of:

$$=1-\left( \prod_{k=1}^{K} \left( \frac{1}{K} \right)^{\frac{1}{K}} \right)^{2}$$

$$=1-\left( \left( \frac{1}{K} \right)^{\frac{K}{K}} \right)^{2}$$

$$=1-\left( \frac{1}{K} \right)^{2}$$

$$=\frac{K^{2}}{K^{2}}-\frac{1}{K^{2}}$$

$$=\frac{K^{2}-1}{K^{2}}$$

# Implications of estimating $\boldsymbol{R}_{\boldsymbol{CS}\boldsymbol{\_}\boldsymbol{adj}}^{\mathbf{2}}$ through $\boldsymbol{R}_{\mathbf{Nagelkerke}}^{\mathbf{2}}\mathbf{=0.15}$ on criterion (ii)

Criterion (ii) holds if equation (22) holds. Let $R_{\mathrm{Nagelkerke}}^{2}$ represent the optimism adjusted $R_{\mathrm{Nagelkerke}}^{2}$then equation (22) simplifies to:

$$S_{VH\_MN}\geq\frac{R_{\mathrm{Nagelkerke}}^{2}*\max\left( R_{CS\_app}^{2} \right)}{R_{\mathrm{Nagelkerke}}^{2}*\max\left( R_{CS\_app}^{2} \right)+\delta*\max\left( R_{CS\_app}^{2} \right)}$$

$$S_{VH\_MN}\geq\frac{R_{\mathrm{Nagelkerke}}^{2}}{R_{\mathrm{Nagelkerke}}^{2}+\delta}$$

If we assume $R_{\mathrm{Nagelkerke}}^{2}=0.15$ when defining $R_{CS\_adj}^{2}$, the right hand side of this equation $=0.75$ and this criterion will always hold when $S_{VH\_MN}$ is targeted at a threshold of $0.9$. This is unlikely to pose an issue as $R_{\mathrm{Nagelkerke}}^{2}=0.15$ is a very safe assumption leading to high sample sizes, but it does make criterion (ii) obsolete. This is important for multinomial logistic regression as we have not been able to make any recommendations on how to estimate $R_{CS\_adj}^{2}$, except through the assumption $R_{\mathrm{Nagelkerke}}^{2}=0.15$. It provides extra weight to the recommendation that researchers report $R_{CS}^{2}$ estimates of multinomial models when fitting them.

# Code for worked example in section 5 of main manuscript

##### This R code will estimate R2_CS for each distinct logistic regression model, following the process of Riley et al (doi: 10.1002/sim.8806)

##### The C statistic values on which the calculation will be based, are extracted from van Calster et al (doi: 10.1186/1471-2288-10-96)

set.seed(101)

### Load relevant packages

library(pROC)

###################################################################################

###################################################################################

##### STEP'S 1 and 2: Identify values for Q, p_k, p_k_r, max(R2_CS), R2_CS_adj and

##### R2_CS_adj_k_r

###################################################################################

###################################################################################

### First define the number of events in each category

EV1 <- 2557 # benign

EV2 <- 186 # Borderline

EV3 <- 176 # Stage 1

EV4 <- 467 # Stage 2 - 4

EV5 <- 120 # Metastatic

############

### Define Q

############

Q <- 17

########################

### Define p_k and p_k_r

########################

## Define total number of events

n.total <- EV1 + EV2 + EV3 + EV4 + EV5

## Calculate p_k

p.1 <- EV1/n.total

p.2 <- EV2/n.total

p.3 <- EV3/n.total

p.4 <- EV4/n.total

p.5 <- EV5/n.total

p1

p2

p3

p4

p5

## Calculate p_k_r

p.1.2 <- (EV1 + EV2)/n.total

p.1.3 <- (EV1 + EV3)/n.total

p.1.4 <- (EV1 + EV4)/n.total

p.1.5 <- (EV1 + EV5)/n.total

p.2.3 <- (EV2 + EV3)/n.total

p.2.4 <- (EV2 + EV4)/n.total

p.2.5 <- (EV2 + EV5)/n.total

p.3.4 <- (EV3 + EV4)/n.total

p.3.5 <- (EV3 + EV5)/n.total

p.4.5 <- (EV4 + EV5)/n.total

########################

### Calculate max(R2_CS)

########################

max_R2_CS <- 1 - (p.1^p.1*p.2^p.2*p.3^p.3*p.4^p.4*p.5^p.5)^2

max_R2_CS

########################

### Calculate R2_CS_adj

########################

### Calculate an estimte of R2_CS_app, based off R2_NAGEL = 0.15

R2_CS_adj <- 0.15*max_R2_CS

R2_CS_adj

###########################

### Calculate R2_CS_adj_k_r

###########################

### Define the C-statistic values for each model

### These were calculated in a temporal validation, so will give estimates of R2_CS

### that do not need to be adjusted for optimism (i.e. R2_CS_adj)

### The pairwise C-statistic for Benign vs Borderline malignent is 0.85

### The pairwise C-statistic for Benign vs Stage 1 0.92

### The pairwise C-statistic for Benign vs Stage 2 - 4 is 0.99

### The pairwise C-statistic for Benign vs Metastatic is 0.95

C.1.2 <- 0.85

C.1.3 <- 0.92

C.1.4 <- 0.99

C.1.5 <- 0.95

### The pairwise C-statistic for borderline vs Stage 1 is 0.75

### The pairwise C-statistic for borderline vs Stage 2 - 4 is 0.95

### The pairwise C-statistic for borderline vs Metastatic is 0.87

C.2.3 <- 0.75

C.2.4 <- 0.95

C.2.5 <- 0.87

### The pairwise C-statistic for stage 1 vs Stage 2 - 4 is 0.87

### The pairwise C-statistic for stage 1 vs Metastatic is 0.71

C.3.4 <- 0.87

C.3.5 <- 0.71

### The pairwise C-statistic for Stage 2 - 4 vs Metastatic is 0.82

C.4.5 <- 0.82

### Calculate pairwise outcome proportions (phi), of category k relative to category i

phi.1.2 <- EV2/(EV1 + EV2)

phi.1.3 <- EV3/(EV1 + EV3)

phi.1.4 <- EV4/(EV1 + EV4)

phi.1.5 <- EV5/(EV1 + EV5)

phi.2.3 <- EV3/(EV2 + EV3)

phi.2.4 <- EV4/(EV2 + EV4)

phi.2.5 <- EV5/(EV2 + EV5)

phi.3.4 <- EV4/(EV3 + EV4)

phi.3.5 <- EV5/(EV3 + EV5)

phi.4.5 <- EV5/(EV4 + EV5)

phi.1.2

phi.1.3

phi.1.4

phi.1.5

phi.2.3

phi.2.4

phi.2.5

phi.3.4

phi.3.5

phi.4.5

### Create a function to simulate R2 from a C-statistic and pairwise outcome proportion

simulate.R2 <- function(N, prop.in, C.in){

## Create an empty dataset

output.dat <- data.frame(matrix(ncol = 2, nrow = N))

colnames(output.dat) <- c("Y", "LP")

## Create the outcome variable

Y.vec <- rbinom(N, 1, prop.in)

## Create the vector of mean values for the linear predictor data generation

Y.vec.mu <- Y.vec*sqrt(2)*qnorm(C.in, 0, 1)

## Generate the linear predictor

LP.vec <- rnorm(N,Y.vec.mu,1)

## Assign these into an output dataset

output.dat$Y <- as.integer(Y.vec)

output.dat$LP <- LP.vec

## Fit a logistic regression to this dataset

model.out <- glm(Y ~ LP.vec, family = binomial(link = "logit"), data = output.dat)

model.out

## Check the AUC is correct, matches input

#C.stat.sim <- as.numeric(roc(Y ~ LP.vec, data = output.dat)$auc)

## Fit a null model also, to calculate likelihood ratio

model.null <- glm(Y ~ 1, family = binomial(link = "logit"), data = output.dat)

## Calculate likelihood ratio statistics

LR <- as.numeric(-2*(logLik(model.null) - logLik(model.out)))

## Calculate R2_CS_APP

R2_CS_APP <- 1 - exp(-LR/N)

R2_CS_APP

## Output object

return(R2_CS_APP)

}

### Calculate R2_CS_adj.k.i according to the simulation approach of Riley.

R2_CS_adj.1.2 <- simulate.R2(1000000, phi.1.2, C.1.2)

R2_CS_adj.1.3 <- simulate.R2(1000000, phi.1.3, C.1.3)

R2_CS_adj.1.4 <- simulate.R2(1000000, phi.1.4, C.1.4)

R2_CS_adj.1.5 <- simulate.R2(1000000, phi.1.5, C.1.5)

R2_CS_adj.2.3 <- simulate.R2(1000000, phi.2.3, C.2.3)

R2_CS_adj.2.4 <- simulate.R2(1000000, phi.2.4, C.2.4)

R2_CS_adj.2.5 <- simulate.R2(1000000, phi.2.5, C.2.5)

R2_CS_adj.3.4 <- simulate.R2(1000000, phi.3.4, C.3.4)

R2_CS_adj.3.5 <- simulate.R2(1000000, phi.3.5, C.3.5)

R2_CS_adj.4.5 <- simulate.R2(1000000, phi.4.5, C.4.5)

R2_CS_adj.1.2

R2_CS_adj.1.3

R2_CS_adj.1.4

R2_CS_adj.1.5

R2_CS_adj.2.3

R2_CS_adj.2.4

R2_CS_adj.2.5

R2_CS_adj.3.4

R2_CS_adj.3.5

R2_CS_adj.4.5

###########################

###########################

##### STEP 3: Criterion (i)

###########################

###########################

## Let S be the level of shrinkage we are targeting

S <- 0.9

## Calculate m_k_r

m.1.2 <- Q/((S - 1)*log(1 - R2_CS_adj.1.2/S))

m.1.3 <- Q/((S - 1)*log(1 - R2_CS_adj.1.3/S))

m.1.4 <- Q/((S - 1)*log(1 - R2_CS_adj.1.4/S))

m.1.5 <- Q/((S - 1)*log(1 - R2_CS_adj.1.5/S))

m.2.3 <- Q/((S - 1)*log(1 - R2_CS_adj.2.3/S))

m.2.4 <- Q/((S - 1)*log(1 - R2_CS_adj.2.4/S))

m.2.5 <- Q/((S - 1)*log(1 - R2_CS_adj.2.5/S))

m.3.4 <- Q/((S - 1)*log(1 - R2_CS_adj.3.4/S))

m.3.5 <- Q/((S - 1)*log(1 - R2_CS_adj.3.5/S))

m.4.5 <- Q/((S - 1)*log(1 - R2_CS_adj.4.5/S))

### Calculate n_k_r for criterion (i) for each submodel

N_C1.1.2 <- m.1.2/p.1.2

N_C1.1.3 <- m.1.3/p.1.3

N_C1.1.4 <- m.1.4/p.1.4

N_C1.1.5 <- m.1.5/p.1.5

N_C1.2.3 <- m.2.3/p.2.3

N_C1.2.4 <- m.2.4/p.2.4

N_C1.2.5 <- m.2.5/p.2.5

N_C1.3.4 <- m.3.4/p.3.4

N_C1.3.5 <- m.3.5/p.3.5

N_C1.4.5 <- m.4.5/p.4.5

N_C1.1.2

N_C1.1.3

N_C1.1.4

N_C1.1.5

N_C1.2.3

N_C1.2.4

N_C1.2.5

N_C1.3.4

N_C1.3.5

N_C1.4.5

### Take the ceiling of the maximum of these as the sample size for criteiron (i)

N_C1 <- ceiling(max(N_C1.1.2, N_C1.1.3, N_C1.1.4, N_C1.1.5, N_C1.2.3, N_C1.2.4, N_C1.2.5,

N_C1.3.4, N_C1.3.5, N_C1.4.5))

N_C1

### Now calculate number of each event we expect to see in a datast of this size

N_C1*p.1

N_C1*p.2

N_C1*p.3

N_C1*p.4

N_C1*p.5

############################

############################

##### STEP 4: Criterion (ii)

############################

############################

N_C2 <- 4*Q/((R2_CS_adj/(R2_CS_adj + 0.05*max_R2_CS) - 1)*log(1 - R2_CS_adj - 0.05*max_R2_CS))

N_C2 <- ceiling(N_C2)

N_C2

### Now calculate number of each event we expect to see in a datast of this size

N_C2*p.1

N_C2*p.2

N_C2*p.3

N_C2*p.4

N_C2*p.5

#############################

#############################

##### STEP 5: Criterion (iii)

#############################

#############################

N_C3.1 <- qchisq(1-0.05/5, 1)*p.1*(1-p.1)/0.05^2

N_C3.2 <- qchisq(1-0.05/5, 1)*p.2*(1-p.2)/0.05^2

N_C3.3 <- qchisq(1-0.05/5, 1)*p.3*(1-p.3)/0.05^2

N_C3.4 <- qchisq(1-0.05/5, 1)*p.4*(1-p.4)/0.05^2

N_C3.5 <- qchisq(1-0.05/5, 1)*p.5*(1-p.5)/0.05^2

N_C3.1

N_C3.2

N_C3.3

N_C3.4

N_C3.5

N_C3 <- ceiling(max(N_C3.1, N_C3.2, N_C3.3, N_C3.4, N_C3.5))

N_C3

### Now calculate number of each event we expect to see in a datast of this size

N_C3*p.1

N_C3*p.2

N_C3*p.3

N_C3*p.4

N_C3*p.5

#####################################################################

#####################################################################

##### STEP 6: Take the maximum sample size across all three criteria

#####################################################################

#####################################################################

N_C1

N_C2

N_C3

N <- max(N_C1, N_C2, N_C3)

N

# References

1. Morris TP, White IR, Crowther MJ. Using simulation studies to evaluate statistical methods. *Stat Med* 2019; 38: 2074–2102.

2. R Core Team. R: A Language and Environment for Statistical Computing. *R Foundation for Statistical Computing*, https://www.r-project.org/ (2020).

3. RStudio: Integrated Development for R. RStudio Team, http://www.rstudio.com/ (2020).

4. Yee TW. The VGAM Package for The VGAM Package for Categorical Data Analysis. *J Stat Softw* 2010; 32: 1–34.

5. Microsoft Corporation and Steve Weston (2020). foreach: Provides Foreach Looping Construct. R package version 1.5.1. https://CRAN.R=project.org/package=foreach.

6. Microsoft Corporation and Steve Weston (2020). doParallel: Foreach Parallel Adaptor for the ‘parallel’ Package. R package version 1.0.16. https://CRAN.R-project.org/package=doParallel.

7. Pate A. GitHub repository: MRC-Multi-Outcome-Project-8-Multinomial-Sample-Size, https://github.com/alexpate30/MRC-multi-outcome/tree/main/Project 8 Multinomial Sample Size (2021).

8. Hoorde K Van, Vergouwe Y, Timmerman D, et al. Assessing calibration of multinomial risk prediction models. *Stat Med* 2014; 33: 2585–2596.

9. Steyerberg EW. *Clinical Prediction Models - A Practical Approach to Development, Validation, and Updating*. Springer, 2009.

10. Harrell FE. *Regression Modeling Strategies*. Springer S. Cham, Switzerland: Springer, 2015.

11. Van Calster B, Nieboer D, Vergouwe Y, et al. A calibration hierarchy for risk models was defined: From utopia to empirical data. *J Clin Epidemiol* 2016; 74: 167–176.

12. Steyerberg EW, Vergouwe Y. Towards better clinical prediction models : seven steps for development and an ABCD for validation. *Eur Heart J* 2014; 35: 1925–1931.

13. Agresti A. *Categorical Data Analysis*. Wiley series: United States of America, 2002.

14. de Jong VMT, Eijkemans MJC, Van Calster B, et al. Sample size considerations and predictive performance of multinomial logistic prediction models. *Stat Med* 2019; 38: 1601–1619.

15. Ensor J, Martin EC, Riley RD. pmsampsize: Calculates the Minimum Sample Size Required for Developing a Multivariable Prediction Model. R package version 1.0.3, https://cran.r-project.org/web/packages/pmsampsize/index.html (2020).

16. Calster B Van, Smeden M Van, Cock B De, et al. Regression shrinkage methods for clinical prediction models do not guarantee improved performance: Simulation study. *Stat Methods Med Res* 2020; 29: 3166–3178.

17. Houwelingen V, Cessie L, Regression R. Shrinkage and penalized likelihood as methods to improve predictive accuracy. *Stat Neerl* 2001; 55: 17–34.

18. Martin GP, Riley RD, Collins GS, et al. Developing Clinical Prediction Models when adhering to minimum sample size recommendations: the importance of quantifying bootstrap variability in tuning parameters and predictive performance. *Stat Methods Med Res (In Press*.

19. Riley RD, Snell KIE, Martin GP, et al. Penalization and shrinkage methods produced unreliable clinical prediction models especially when sample size was small. *J Clin Epidemiol* 2021; 132: 88–96.

20. Christodoulou E, van Smeden M, Edlinger M, et al. Adaptive sample size determination for the development of clinical prediction models. *Diagnostic Progn Res*; 5. Epub ahead of print 2021. DOI: 10.1186/s41512-021-00096-5.
